# Red-Light-Activatable AND-Gated Antitumor Immunosuppressant

**DOI:** 10.3390/cells12192351

**Published:** 2023-09-26

**Authors:** Ziqi Zhou, Yan Zhang, Simin Xia, Xi Chen

**Affiliations:** 1 Laboratory of Chemical Biology and Frontier Biotechnologies, The HIT Center for Life Sciences (HCLS), Harbin Institute of Technology (HIT), Harbin 150001, China; zzq2000dd@163.com (Z.Z.); 22s028046@stu.hit.edu.cn (Y.Z.);; 2 School of Life Science and Technology, Harbin Institute of Technology (HIT), Harbin 150001, China

**Keywords:** antitumor immunosuppressant, FTY720, logic gate, BODIPY photocage, red light, photodynamic therapy

## Abstract

Immunosuppressants are emerging as promising candidates for cancer therapy with lower cytotoxicity compared to traditional chemotherapy drugs; yet, the intrinsic side effects such as immunosuppression remain a critical concern. Herein, we introduce a photoactivatable antitumor immunosuppressant called dmBODIPY-FTY720 (BF) that shows no cytotoxicity but can be temporally and locally activated by deep-red light illumination to induce tumor cell apoptosis. To further reduce potential side effects, we integrate BF with another classic photosensitizer called methylene blue (MB) that is activated under the same wavelength of deep-red light (>650 nm) and successfully establish a red-light-activatable AND Boolean logic gate through a mechanism that we found to be synergetic apoptotic induction. At further decreased dosages, deep-red light illumination does not induce cell death in the presence of either BF or MB, but significant cancer cell death is triggered in the presence of both drugs. Therefore, the dosage of BF is further reduced, which will be highly beneficial to minimize any potential side effects of BF. This AND-gated strategy has been successfully applied in vivo for effective suppression of hepatocarcinoma tumors in living mice.

## 1. Introduction

Recently, immunosuppressants have emerged as a new class of drugs that exhibit antitumor effects [1,2]. Representative candidates include rapalogs such as rapamycin and everolimus [1,3], fingolimod (FTY720) [4], prodigiosins (PrGs) [5], capecitabine [6], and others. Compared to traditional chemotherapy drugs, such as cis-platin and others, which possess strong cytotoxicity [7,8], immunosuppressants have significantly reduced toxicity [9,10]. FTY720 is a clinically approved immunosuppressant [11] and has recently been shown to exert efficient antitumor activity [4]. Among known anticancer immunosuppressants, the detailed molecular mechanism of FTY720 was recently dissected via structural biological approaches [12]; additionally, FTY720 is fairly inexpensive (~100 USD/g, Aladdin), easily accessible from commercial vendors, and its anticancer activity has been well evaluated. Given these factors, the rational design of a photoactivatable FTY720 for phototherapy is an appealing option.

Phototherapy, which employs light illumination, is another form of cancer treatment that is characterized by the ability to locally and temporally induce cell death with minimal systemic toxicity [13,14]. Red light, in particular at the longer wavelength end that falls into the therapeutic window, has a significant advantage in terms of tissue-penetrating and being free of phototoxicity compared to shorter wavelengths of light such as blue and ultraviolet light [15,16]. Therefore, we envision that a deep-red-light-activatable antitumor immunosuppressant will endow the combined advantages of low cytotoxicity and reduced systemic toxicity with the help of localized photoactivation.

In order to further reduce potential side effects [17], it would be necessary to reduce the dosage of immunosuppressant to an even greater level while still maintaining sufficient antitumor efficacy. For this, we plan to construct a red-light-operated AND Boolean logic gate [18,19] in which the presence of only one drug would not produce any output unless both drugs are present simultaneously. Methylene blue (MB) is a drug used in photodynamic therapy that generates singlet reactive oxygen species (ROS) upon red light activation [20]. In recent years, MB has been explored to act synergistically with chemotherapy drugs, such as the widely used paclitaxel drug [21] or in the so-called chemo-photodynamic therapy modality [22], to dramatically enhance the antitumor efficacies. More importantly, MB demonstrates a photoactivation wavelength in the far-red range [20], which aligns effectively with the far-red-light-activatable FTY720 that we intend to develop. Therefore, we envisioned that it might be possible to integrate photosensitive MB with the photoactivatable FTY720 to establish the first red-light-operated AND logic gate as a next-generation photo/photo-therapy modality. In this way, the dosages of both drugs could be set at an even safer level, securing reduced potential side effects from these drugs.

## 2. Experimental Methods

### 2.1. General

All chemicals and reagents are commercially available, including those for organic synthesis, cell culture, and various assays, and are used without further treatment unless otherwise mentioned. The experimental section and more detailed methods are provided in the Supporting Information, including the synthesis and preparation of the dmBODIPY-FTY720 probe, CCK-8 assay, HPLC analysis, confocal microscopy, UV–vis and fluorescence spectroscopic analysis, measurement of ROS, flow cytometry, etc. 

### 2.2. Organic Synthesis

Unless otherwise specified, all chemicals were purchased from commercial vendors and were used without further purification. The ^1^H- and ^13^C-NMR spectra were measured on a 600 MHz Bruker BioSpin GmbH magnetic resonance spectrometer. Data for ^1^H-NMR spectra are reported as follows: Chemical shifts are reported as δ in units of parts per million (ppm); multiplicities are reported as follows: s (singlet), d (doublet), t (triplet), q (quartet), dd (doublet of doublets), m (multiplet), or br (broadened); coupling constants are reported as J values in Hertz (Hz); the number of protons (n) for a given resonance is indicated as nH and is based on the spectra integration values. High-resolution mass spectra (HR-MS) measurements were performed using electron spray ionization (ESI) through customer service.

### 2.3. Ethical Statement

The mice used in this study were maintained under specific pathogen-free (SPF) conditions and handled in accordance with the guidelines set by the Institutional Animal Care and Use Committee (IACUC) of the Harbin Institute of Technology. The study was conducted with the approval of the IACUC, with the permit number IACUC-2021052. The mice were housed in a controlled environment with a 12 h light/12 h dark cycle, a temperature of 24 ± 2 °C, and humidity maintained at 50 ± 10%. They were provided with autoclaved chow and water. The IACUC has set a limit of 20 mm for tumor size, or 10% of the body weight, and this limit was not exceeded in our study.

### 2.4. Generation of Xenograft Mouse Model

Immunodeficient BALB/c nude female mice, aged 4–6 weeks, were obtained from Beijing Vital River Laboratory Animal Technology Co., Ltd. (Beijing, China). HepG2 cells were cultured in a standard Φ ~85 mm Petri dish until they reached the exponential phase. The study design did not consider the sex of the mice, as hepatocarcinoma is not a gender-based disease. Subsequently, the HepG2 cells were washed with 10 mL of PBS and treated with 1 mL of trypsin for 5–10 min to ensure complete detachment from the growing surface. Afterward, 3 mL of PBS was added to suspend the detached cells. The cell suspension was then centrifuged at 800× *g* for 8 min at 4 °C. The clear supernatant was discarded, and the cell pellets were resuspended in a freshly prepared ice-cold mixture of PBS/Matrigel (Cat# M8370, Solarbio, Beijing, China) in a 1:1 (*v*/*v*) ratio on ice. The final cell density was approximately 5 × 10^7^ mL^−1^. To establish the HepG2 xenograft mouse model, approximately 5 × 10^6^ HepG2 cells in 0.1 mL of PBS/Matrigel solution were subcutaneously injected into the axillary region of the BALB/c nude mice. Tumors typically appeared within 1–2 weeks and continued to grow steadily.

### 2.5. Red Light AND-Gated Suppression of Tumor Growth In Vivo

Drug solutions in DMSO were injected intratumorally from different directions to ensure even distribution within the tumor. Unless otherwise specified, approximately 20 μL of dmBODIPY-FTY720 in DMSO (10.8 mg∙mL^−1^, final concentration ~12 mg∙kg^−1^), approximately 20 μL of MB in DMSO (1.8 mg∙mL^−1^, final ~2 mg∙kg^−1^ body weight), approximately 20 μL of dmBODIPY-FTY720 (10.8 mg∙mL^−1^) combined with MB (1.8 mg∙mL^−1^) in DMSO ([1, 1] AND input), or 20 μL of DMSO (blank, or [0, 0] input) were administered intratumorally each time. Subsequently, the tumor region was exposed to 650 nm red light for 60 min, followed by another 60 min of red light exposure the next day. This injection–illumination–illumination procedure was repeated every two days. Tumor size data were measured daily using a caliper [Φ = (Φ_L_ + Φ_S_)/2], and tumor volumes were calculated using the equation: V = 1/6(πΦ^3^).

## 3. Results and Discussion

### 3.1. Design, Preparation and Spectroscopic Characterization of Red Light Activatable FTY720, dmBODIPY-FTY720

**Figure 1:**
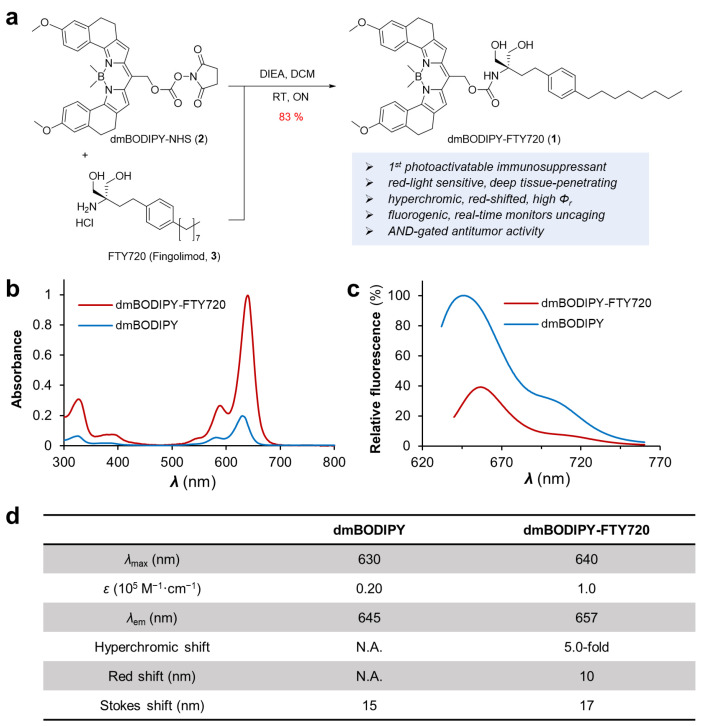
Synthesis and spectroscopic analysis of dmBODIPY-FTY720 (**1**, BF). (**a**) Synthetic scheme for the preparation of **1**. Abbreviations: DIEA, *N*,*N*-diisopropylethylamine; DCM, dichloromethane; RT, room temperature; ON, overnight. (**b**) UV-vis absorption spectra of dmBODIPY (10 μM) and dmBODIPY-FTY720 (10 μM) recorded at room temperature (RT) in pH 7.2 PBS buffer. (**c**) Fluorescence spectra of dmBODIPY (10 μM) and dmBODIPY-FTY720 (10 μM) recorded at RT in pH 7.2 PBS buffer. (**d**) Summary of key photophysical properties of dmBODIPY-OH and **1**. N.A., not applicable.

Recent advancements in photocage chemistry have led to the identification of new classes of photocages that are liable upon red light illumination, for example, *B*,*B*-dimethyl substituted BODIPY (or dmBODIPY) [23]. The substitution of the two fluorine atoms at the boron core of a traditional difluoro-BODIPY could further enhance the photouncaging quantum yield through the blocking of unproductive conical intersections [23]. On the other hand, structure–activity relationship studies and other works reveal that the amino group on FTY720 is essential for its biological function; for example, the recent crystallographic structure of FTY720 in complex with sphingosine-1-phosphate (S1P) receptor revealed that the amino group of this drug is positioned inward the binding tunnel of the S1P receptor [12]. Therefore, we envisioned that photocage of FTY720 at its amino group by dmBODIPY photocage can result in a deep red-light-responsive photo-immunosuppressant anticancer drug with reasonable uncaging quantum efficiency. Hence, we designed and prepared dmBODIPY-FTY720 (**1**), or BF (Figure 1). We first prepared the *N*-hydroxysuccinimide (NHS) ester derivative of dmBODIPY, denoted as dmBODIPY-NHS (**2**), through multi-step organic synthesis. dmBODIPY-NHS (**2**) is a general-purpose amine-reactive NHS ester form of dmBODIPY photocage, which can quickly react with FTY720 (**3**) to create **1**.

According to UV-Vis spectroscopic analysis, **1** shows a significantly higher absorbance (*ε* = 1.0 × 10^5^ M^−1^∙cm^−1^) than dmBODIPY (ε = 0.20 × 10^5^ M^−1^∙cm^−1^) photocage, suggesting a strong hyperchromic shift, which is valuable in terms of enhancing photouncaging efficiency due to increased absorptivity. Furthermore, **1** exhibits a maximal absorption wavelength of 657 nm with a 10 nm red shift compared to dmBODIPY photocage, hence benefiting photoactivation using a relatively longer wavelength of red light. We also found that the fluorescence of the dmBODIPY cage is significantly higher than **1**, which may facilitate monitoring of the photouncaging process via the detection of fluorescence enhancement [24]. For example, it may allow tracking of photo-activatable drug release in vivo, which would be otherwise difficult for other photoactivatable drugs. Hence, we have generated a deep red-light-sensitive photocage-tethered immunosuppressant drug with favorable photophysical properties, including hyperchromic enhancement, red shift, and fluorogenicity during photouncaging.

### 3.2. dmBODIPY-FTY720 (***1***) Is Readily Cell-Permeable and Releases FTY720 upon Deep Red-Light Illumination

**Figure 2:**
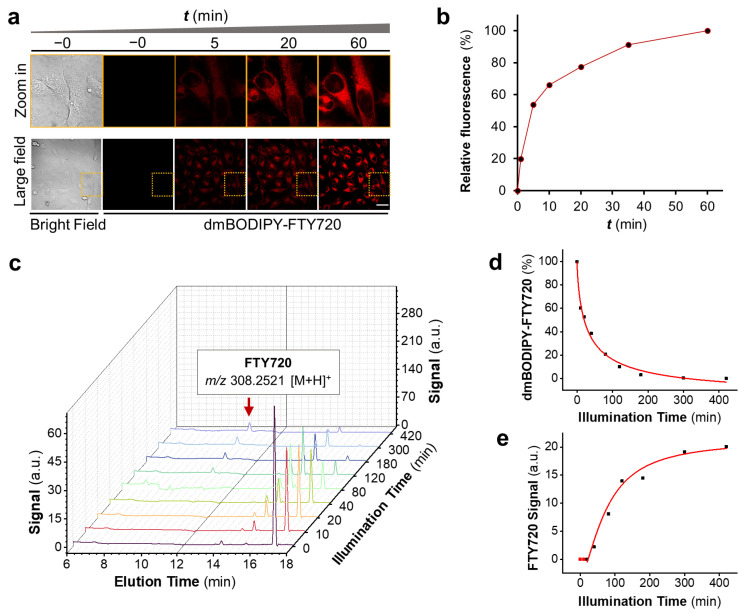
dmBODIPY-FTY720 (**1**) is highly cell permeable and releases FTY720 upon red light illumination. (**a**) **1** rapidly enters into live HeLa cells in a time scale of minutes. A concentration of 10 μM **1** in cell culture medium was applied, and confocal micrographs were taken to visualize its cellular uptake. Dotted boxes indicate the zoom in area; scale bar: 50 μm. (**b**) Quantification of cell fluorescence enhancement over time was performed. (**c**) The waterfall plot of HPLC chromatographs reveals the decomposition of **1** and the formation of FTY720 product along with increased uncaging time; HPLC chromatographs of different time points were depicted in different colors for clarity. The peak at *t*_R_ 17.0 min (λ = 640 nm) represents **1** while the peak at *t*_R_ 10.2 min (λ = 265 nm) indicates uncaged FTY720 (*m*/*z* 308.2521 [M + H]^+^). (**d**) Quantification of the remained percentage of **1** along time. (**e**) Quantification of the generation of **1** along time.

With **1** in hand, we studied if this molecule is cell permeable or not. This is a critical parameter because FTY720 needs to be phosphorylated by sphingosine kinase 1 (SphK1) inside live cells to convert itself to a phosphorylated form [25] for exerting its biological function, such as responding to apoptotic stimuli. For this, 10 μM of dmBODIPY-FTY720 was added to the cell culture medium, and the cellular uptake of this drug was readily visualized thanks to the intrinsic deep-red light fluorescence of this molecule. As can be seen from Figure 2a, **1** is readily cell permeable, and it starts to enter live cells within a few minutes; soon, a considerable amount of drug enters live cells after half an hour (Figure 2b). This result also reveals that tethering of the dmBODIPY cage enhances the bioavailability of FTY720 because FTY720 is known to be poorly water soluble [26], which strongly hampers its bioavailability.

Next, we studied whether FTY720 was released from **1** upon red light illumination. We performed an HPLC analysis using solutions of dmBODIPY-FTY720 irradiated with increasing dosages of 650 nm light. Since **1** (λ_max_ 640 nm) and FTY720 (λ_max_ 265 nm) exhibit significantly different maximal absorption wavelengths, UV–vis detection in HPLC was set at both 265 nm and 640 nm, and the resultant two HPLC chromatographs were combined (λ = 265 nm for *t*_R_ < 11 min segment; λ = 640 nm for *t*_R_ > 12 min segment) to facilitate analysis. HPLC waterfall chromatographs reveal that **1** (*t*_R_ 17.0 min) was rapidly decomposed along with the generation of FTY720 (*t*_R_ 10.2 min) upon red light illumination (Figure 2c). The identity of the peak at *t*_R_ 10.2 min was further confirmed to be FTY720 via high-resolution mass spectrometry analysis (*m*/*z* = 308.2521 [M + H]^+^) (Figure S1). It needs to be noted that the uncaged intermediates seem to undergo further changes (e.g., *t*_R_ 14 min peak), revealing a more complicated photouncaging mechanism. Noteworthy, we also found that the fluorescence of **1** is enhanced upon light illumination (Figure S2), which potentially provides a useful means for real-time monitoring of the photo-release process. Hence, **1** releases FTY720 upon deep red-light illumination, demonstrating that **1** is a red-light-activatable immunosuppressant.

### 3.3. dmBODIPY-FTY720 (***1***) Kills Tumor Cells Only upon Red Light Illumination

**Figure 3:**
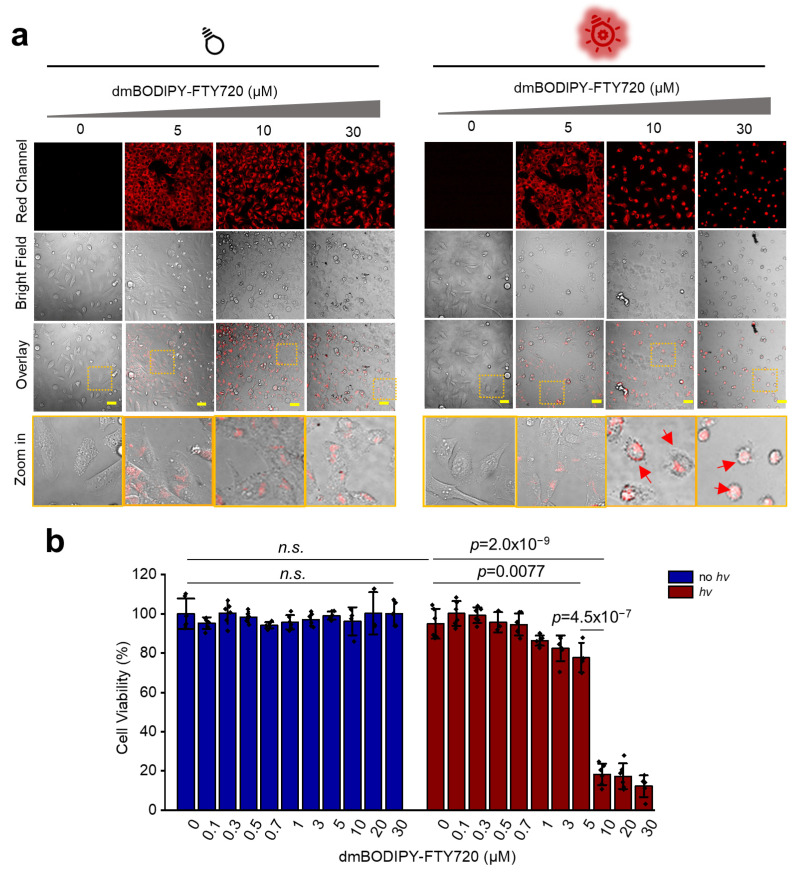
dmBODIPY-FTY720 (**1**) kills cancer cells only upon deep-red light illumination. (**a**) Based on confocal micrographs, **1** does not show apparent toxicity to live HeLa cells even at a concentration as high as 30 μM; however, apparent cell death was observed (red arrows) when 10 μM or higher concentrations of **1** were added with deep-red light illumination (650 nm, 45 min). Dotted boxes indicate the zoom in area; scale bar: 50 μm. (**b**) Statistical cell counting kit-8 (CCK8) cytotoxicity assay revealed that **1** is not cytotoxic without light illumination, while dramatically increased cytotoxicity was observed when 10 μM or higher concentrations were applied with deep-red light (650 nm) illumination; n.s., non-significant.

Next, we determined whether or not **1** could kill cancer cells upon red-light illumination. We used the CCK-8 cytotoxicity assay by adding increasing concentrations of **1** into live HeLa cells. From as low as nanomolar concentration to as high as 30 μM, **1** showed no detectable cytotoxicity, which suggests that **1** is generally safe (Figure 3a,b, left). We then treated live HeLa cells with increased dosages of **1** and subjected them to 650 nm light illumination for 45 min. According to both confocal micrographs and CCK-8 assay results, **1** showed no or non-apparent cytotoxicity at concentrations of 5 μM or below. However, a sharp enhancement of cytotoxicity was observed when 10 μM or higher concentrations of **1** were used (Figure 3a,b, right). Confocal microscopic images also revealed that cells turned into a highly abnormal rounded profile, which is a typical phenomenon for induced cell death [27]. To confirm whether the released FTY720 kills cancer cells or not, we also performed a CCK-8 assay which revealed that FTY720 does kill live HeLa cells (Figure S3). Hence, **1** is a photoactivatable immunosuppressant that kills cancer cells upon deep red-light illumination.

### 3.4. Design of AND-Gated System for Effective Killing of Cancer Cells Using Red Light at Further Reduced Drug Dosages

**Figure 4:**
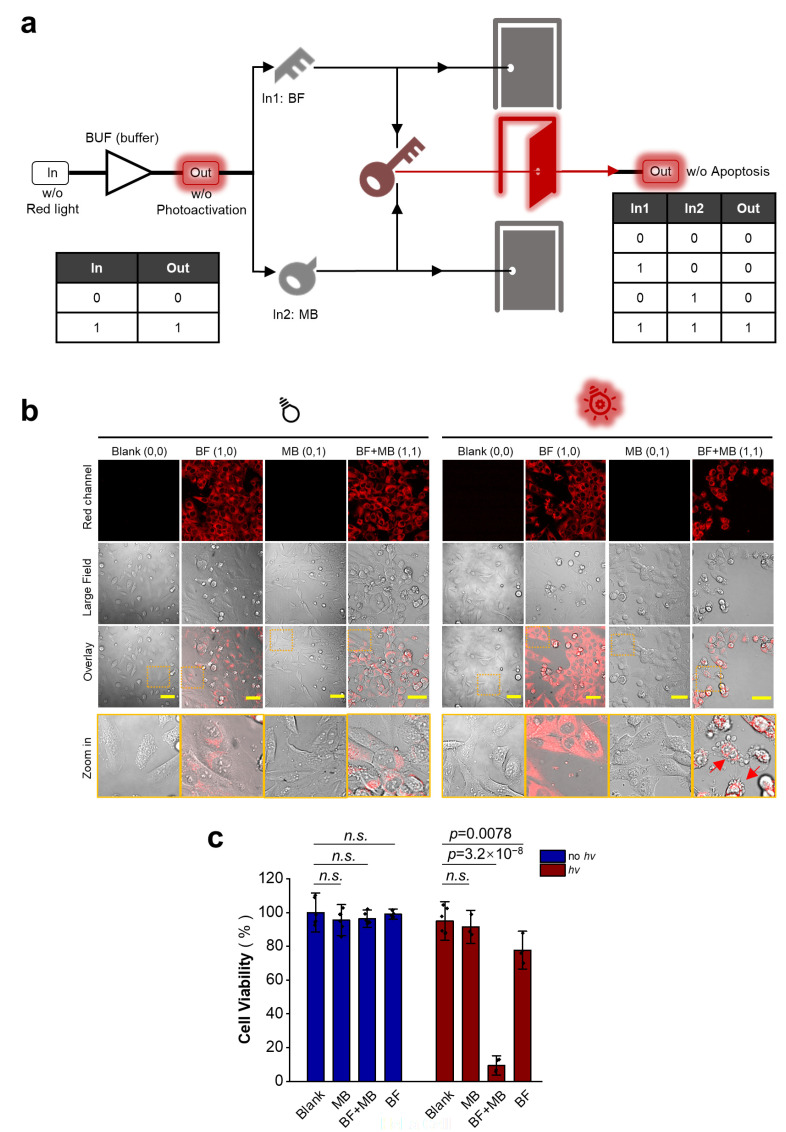
Red light AND gate-operated induction of tumor cell death. (**a**) We designed a red light triggered AND logic gate that is able to induce tumor cell death only with the input of both dmBODIPY-FTY720 (BF) and MB drugs. (**b**) According to confocal micrographs, red light (650 nm) induces HeLa cell death (red arrows) only in the presence of both BF (5 μM) and MB (2 μM) drugs, not only one or none of them. Dot boxes indicate zoom in area; scale bar: 50 μm. (**c**) A CCK-8 assay revealed that red light kills HeLa cells in the presence of both drugs but not only one or none of them, demonstrating the successful construction of a red light AND logic gate. n.s., non-significant.

The results suggest that **1** shows strong red-light-triggered cytotoxicity at 10 μM concentration and above, while at 5 μM or below there is considerably lower cytotoxicity. Due to the concern of potential side effects, it is desirable to establish an AND-gated system in which even lower dosages of this drug can be used while still retaining sufficient antitumor activity. Methylene blue (MB) is known as a photosensitizer that produces ROS to kill cancer cells upon red light illumination [28] with a wavelength fully matching with **1** photoactivation. Hence, by fine-tuning the concentrations of both drugs, it might be possible to reach a condition where sufficient anticancer phototoxicity is exerted due to the potential synergistic action of both drugs [29,30], while no apparent cytotoxicity would exist in the presence of either one drug or neither. This will essentially create a Boolean AND-gated system that works only in the presence of both drug inputs with red-light illumination as a prerequisite (BUF gate) (Figure 4a).

To check this possibility, we evaluated the red-light-activated cytotoxicity of different concentrations of MB upon 650 nm light illumination. It can be seen that MB alone below 15 μM shows no dark cytotoxicity without light illumination, which suggests that MB is also a safe drug at moderate or low concentrations (Figure S4). On the other hand, with 650 nm light illumination, MB produces significant cytotoxicity from 5 μM and above, while there is much less or no cytotoxicity at 3 μM or below (Figure S4).

Based on the results above, we used a concentration of **1** at 5 μM and a concentration of MB at 2 μM as inputs for designing the photo-AND logic gate. At both concentrations, neither **1** nor MB could effectively kill cells with or without red light illumination, meaning 0 output in the logic gate truth table (Figure 4b,c, left). On the other hand, in the presence of both photo-responsive drugs, strong cytotoxicity was observed upon red light illumination, indicating 1 output in the truth table (Figure 4b,c, right). To further validate this result, we chose another cancer cell line, HepG2, a hepatocarcinoma cell line, and obtained similar results (Figure S5). Hence, we have configured a red-light-triggered AND logic gate system that effectively kills cancer cells in the presence of lower concentrations of two drugs.

### 3.5. Mechanistic Study Reveals Synergistic Apoptosis Induction in Red-Light-Triggered AND-Gated Tumor Cell Death

With the results above, we next asked about the mechanism of red-light AND-gated cancer cell death. For this, we performed a flow cytometry analysis to check whether or not cancer cell death was caused via apoptosis because FTY720 has been reported to induce apoptosis through several pathways [31,32,33], while MB also produces apoptotic cells [28]. As shown in Figure S6a,b, control cells without any drug treatment show live cells that fail to reach the Q4 quadrant (lower left). BF (**1**) alone-treated cells under red light illumination show only a weak apoptotic effect. MB-only-treated cells under red light illumination reveal early-stage apoptotic cells that fail in the Q3 quadrant (lower right). In contrast, for cells treated with both BF and MB and subjected to red light illumination, most cells are converted to late-stage apoptotic dead cells that fail in the Q2 quadrant (upper right). It is worth noting that without red light illumination, no apoptosis occurred for cells treated with one drug or even two, suggesting that this is a red-light-activated photo-logic gate system. To the best of our knowledge, this represents the first deep-red light-triggered logic data for the control of cell function.

We then determined whether or not ROS was generated during AND-gated apoptosis induction because MB has been widely known to generate ROS [20], and in recent years it has been found that FTY720 exerts an antitumor effect through a ROS-dependent mechanism [31,34]. We found that cells treated with BF (1, 0), MB (0, 1), or both BF and MB (1, 1) under red light illumination generated lots of ROS the using 2′,7′-dichlorodihydrofluorescein diacetate (DCFH-DA) ROS sensor (Figure S7). DCFH-DA-treated cells were also subjected to flow cytometry analysis, which further validated the formation of ROS in the AND-gated cell group (Figure S8). Hence, AND-gated apoptosis induction by BF and MB might work through a ROS-related cellular mechanism.

### 3.6. Combination of dmBODIPY-FTY720 (BF, ***1***) and MB Efficiently Suppresses Tumor Growth In Vivo under Red Light Illumination

**Figure 5:**
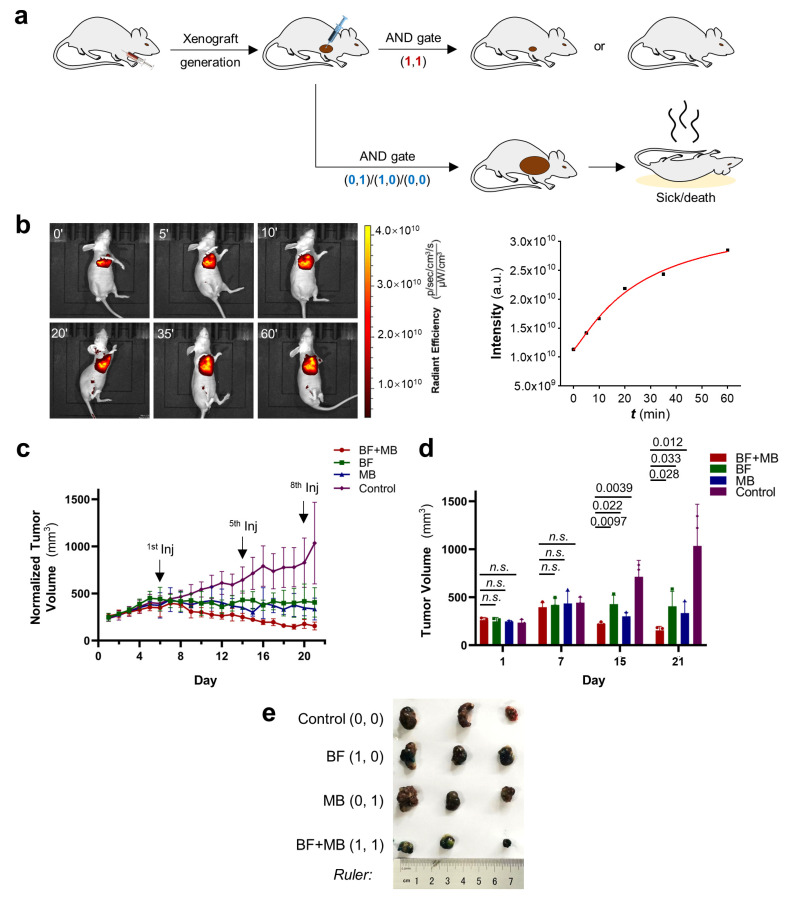
Red-light AND logic-gated suppression of tumor growth in vivo. (**a**) Hepatocarcinoma xenograft mouse models were first generated by subcutaneously injecting about 5 × 10^6^ HepG2 cells into the axillary region of BALB/c nude mice, and the tumors started to grow steadily to around Φ 7–9 mm in diameter before the tumors were subjected to red-light AND-gated treatment. (**b**) Real-time visualization of the fluorescence enhancement (λ_ex_ 620 nm/λ_em_ 670 nm) at the tumor region of a live mouse after intratumoral injection of BF (12 mg∙kg^−1^) and subjected to different durations of 650 nm red light illumination. (**c**) Tumor volumes of MB-injected (2 mg∙kg^−1^), BF-injected (12 mg∙kg^−1^), and simultaneous MB (2 mg∙kg^−1^) and BF (12 mg∙kg^−1^)-injected groups were measured every day. The drugs were intratumorally given and the tumor region was illuminated by 650 nm red light lamp for 60 min; another 60 min of red-light illumination was applied the next day. This injection–illumination procedure was repetitively applied every two days. Error bars: standard deviation (SD) (*n* = 3 mice); one-sided Student’s *t*-test was used. (**d**) Statistical quantification (*n* = 3 mice) of the tumor volume differences at day 1, day 7, day 15, and day 21. n.s., non-significant. (**e**) Tumors derived from euthanized mice of different groups at day 21, which shows that BF + MB (1, 1) treated group carries the smallest tumors.

Finally, we decided to translate this photo-AND logic gate system for the phototherapy of tumors. For this, a hepatocarcinoma xenograft mouse model was first generated using HepG2 cells, and tumors were allowed to grow steadily until around 7–9 mm in diameter before intratumoral injection of the drugs (Figure 5a). Tumors were injected with BF (12 mg∙kg^−1^) alone, MB (2 mg∙kg^−1^) alone, or BF (12 mg∙kg^−1^) and MB (2 mg∙kg^−1^) together. Immediately after giving those drugs, the tumor region was subjected to red light illumination for 60 min. Sixty minutes of red-light illumination in the tumor region was repeated the next day. This phototherapy process was repeated every two days, and the tumor sizes were monitored every day using a caliper.

Interestingly, first, we were able to see that fluorescence of the tumor region steadily increases with increasing dosages of red-light illumination for BF-injected mice (Figure 5b), matching the previously mentioned fluorogenic property of dmBODIPY-FTY720 (Figure S1). Hence, BF is a drug that enables real-time monitoring of the drug release process in vivo during phototherapy. Then, regarding tumor size, it can be observed that mice subjected to phototherapy injected with both drugs show a clear tumor suppression effect, and the tumor size decreases gradually over time. On the other hand, for mice injected with only one drug, tumor growth slows down but is not comparable with the two-drug-injected group (Figure 5c–e). Therefore, this red-light-activatable AND-gated strategy allows efficient suppression of tumor growth in vivo. 

## 4. Conclusions

In summary, we introduced a red-light-activatable AND-gated antitumor immunosuppressant as a modality for killing tumor cells and designed the first red-light-activatable immunosuppressant anticancer drug, dmBODIPY-FTY720 (**1**). The deep-red light (≥650 nm) used falls into the therapeutic window, which facilitates the application of this system in vivo. **1** shows fluorogenicity upon red light activation, thus benefiting real-time monitoring of drug release. In order to further minimize drug dosages, we integrated it with another red-light-sensitive molecule, MB, and established a photo-AND-gated system that is fully operated under red light illumination. Mechanistic study revealed that this AND-gated system probably works through synergistic induction of apoptosis from both MB and FTY720, and the generation of ROS by MB might increase the vulnerability of tumor cells to FTY720 [34].

We successfully translated this photo-AND-gated system for suppression of tumor growth in vivo using the Xenograft mouse model. The results show that the combined injection of both drugs, followed by applying deep red-light illumination, effectively suppressed tumor growth. Nevertheless, there is still potential to further optimize the required drug doses and the frequency of injection/illumination repeat. As the side effects of antitumor immunosuppressants are currently one of the major barriers that prevent their clinic use [4], we envisioned that a synergistic combination between **1** and MB through a red-light AND-gated system may accelerate the introduction of FTY720 to the clinic as an antitumor drug. 

Compared to traditional photodynamic therapy strategies, our AND-gated approach significantly reduces the dosage of PDT drugs, such as the MB used in our study. Additionally, the synergetic strategy produces a more potent effect than a single PDT drug alone in the gated system. Mechanistically, PDT mostly relies on the generation of singlet oxygen [35], which is also harmful to normal cells. In contrast, anticancer immunosuppressants work through different mechanisms to avoid this risk. Based on the discussions above, we therefore expect that the design of new classes of red-light-activatable immunosuppressants, working through a photo-AND-gated system, could become a widely used photo/photo-therapy modality for cancer treatment in the future.

## Data Availability

The data presented in this study are available in this Manuscript and its associated Supplementary Information.

## Supplementary Materials

The following supporting information can be downloaded at: https://www.mdpi.com/article/10.3390/cells12192351/s1, Supporting Information is available and includes supplementary figures, materials and supplementary methods, synthesis and characterization of organic compounds, and NMR spectra. Figure S1: high resolution mass spectrum of FTY720 with tR 10.2 min further validated the identity of FTY720; Figure S2: dmBODIPY-FTY720 is a fluorogenic drug that shows enhanced fluorescence upon red light illumination; Figure S3: CCK-8 cytotoxicity assay of FTY720 at different concentrations in live HeLa cells; Figure S4: methylene blue (MB) shows no or moderate cytotoxicity without red light illumination; whereas upon red light illumination, significant cytotoxicity was detected; Figure S5: AND-gated killing of HepG2 cell; Figure S6: mechanistic study shows that tumor cells undergo apoptosis; Figure S7: ROS was generated upon red light illumination in the presence of BF, MB, or BF+MB group; Figure S8: further validation of ROS generation upon red light illumination in the BF+MB [1, 1] group using flow cytometry analysis. Scheme S1: Synthetic scheme for the preparation of dmBODIPY-FTY720 photocaged immunosuppressant. Abbreviations: DMSO, dimethyl sulfoxide; DCM, dichloromethane; Et3N, triethylamine; DSC, N,N′-disuccinimidyl carbonate; DIEA, N,N-diisopropylethylamine; FTY720, 2-amino-2-[2-(4-octylphenyl)ethyl]propane-1,3-diol hydrochloride.
