# Supplementary material for: Red-Light-Activatable AND-Gated Antitumor Immunosuppressant
Source: Cells. 2023 Sep 26;12(19):2351. doi: 10.3390/cells12192351 (PMC10571834; doi:10.3390/cells12192351)

Supplementary Information

Supplementary Figures

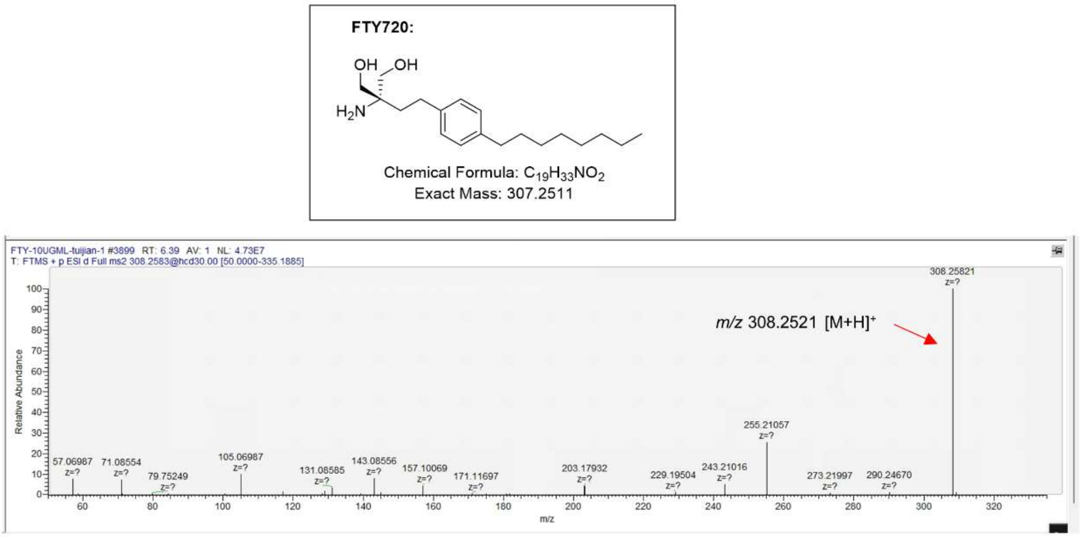

Figure S1. High resolution mass spectrum of FTY720 with t_R 10.2 min further validated the identity of FTY720 (m/z 308.2521 $[M+H]^+$).

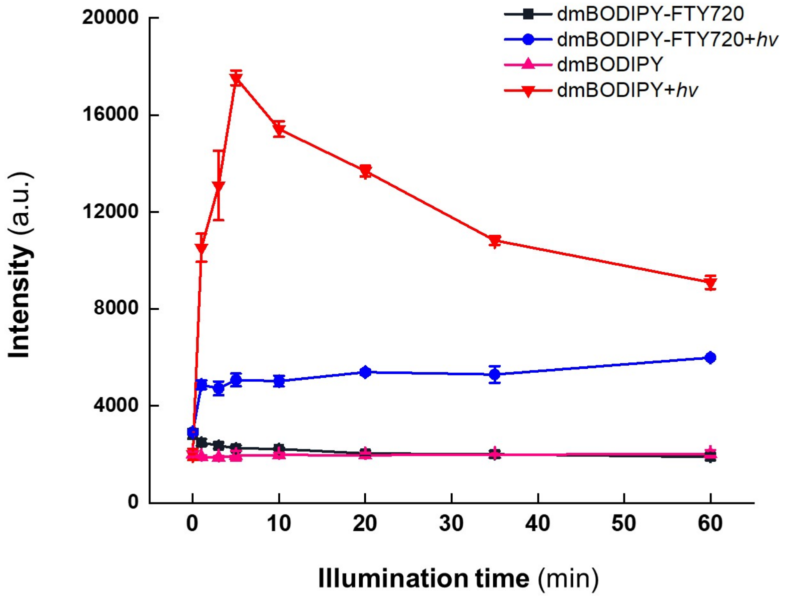

Figure S2. dmBODIPY-FTY720 is a fluorogenic drug that shows enhanced fluorescence upon red light illumination. Without red light illumination, the fluorescence of dmBODIPY-FTY720 is almost consistent (black line) along time; whereas with 650 nm red light illumination, the fluorescence intensity of this molecule increases and then gradually reaches a plateau. The fluorescence enhancement of dmBODIPY cage with or without 650 nm red light illumination was also recorded for comparison.

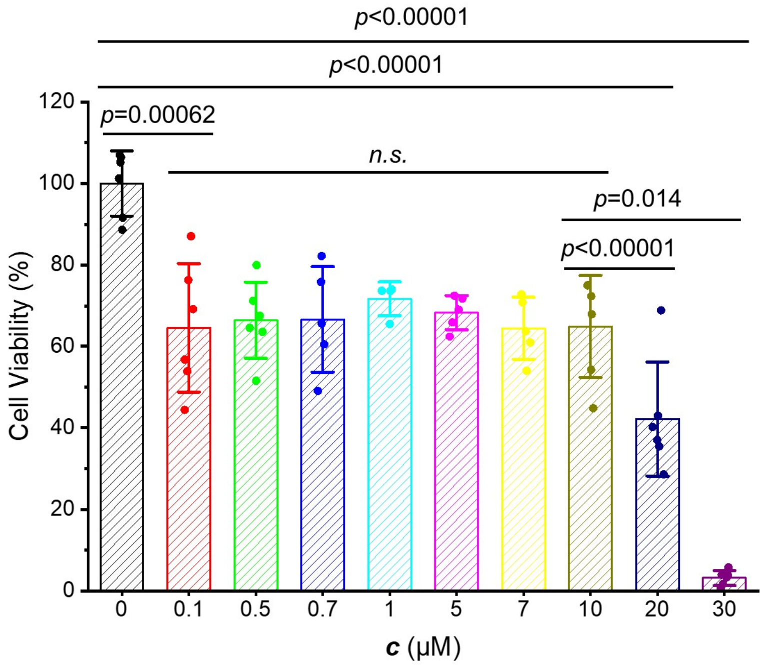

Figure S3. CCK-8 cytotoxicity assay of FTY720 at different concentrations in live HeLa cells.

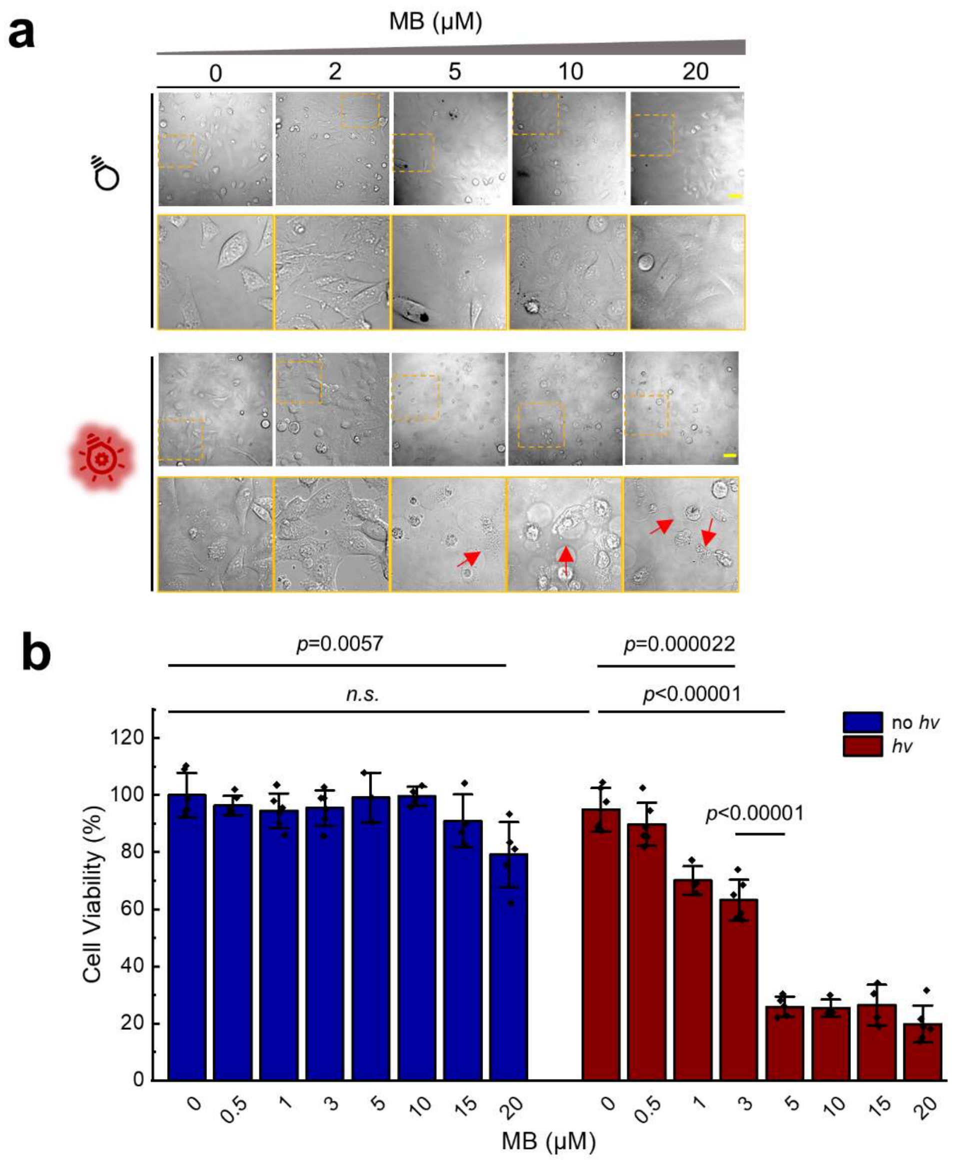

Figure S4. Methylene blue (MB) shows no or moderate cytotoxicity without red light illumination; whereas upon red light illumination, significant cytotoxicity was detected. **a**) Representative confocal microscopic images; scale bars: 50 μm. **b**) Statistic analysis of CCK-8 results.

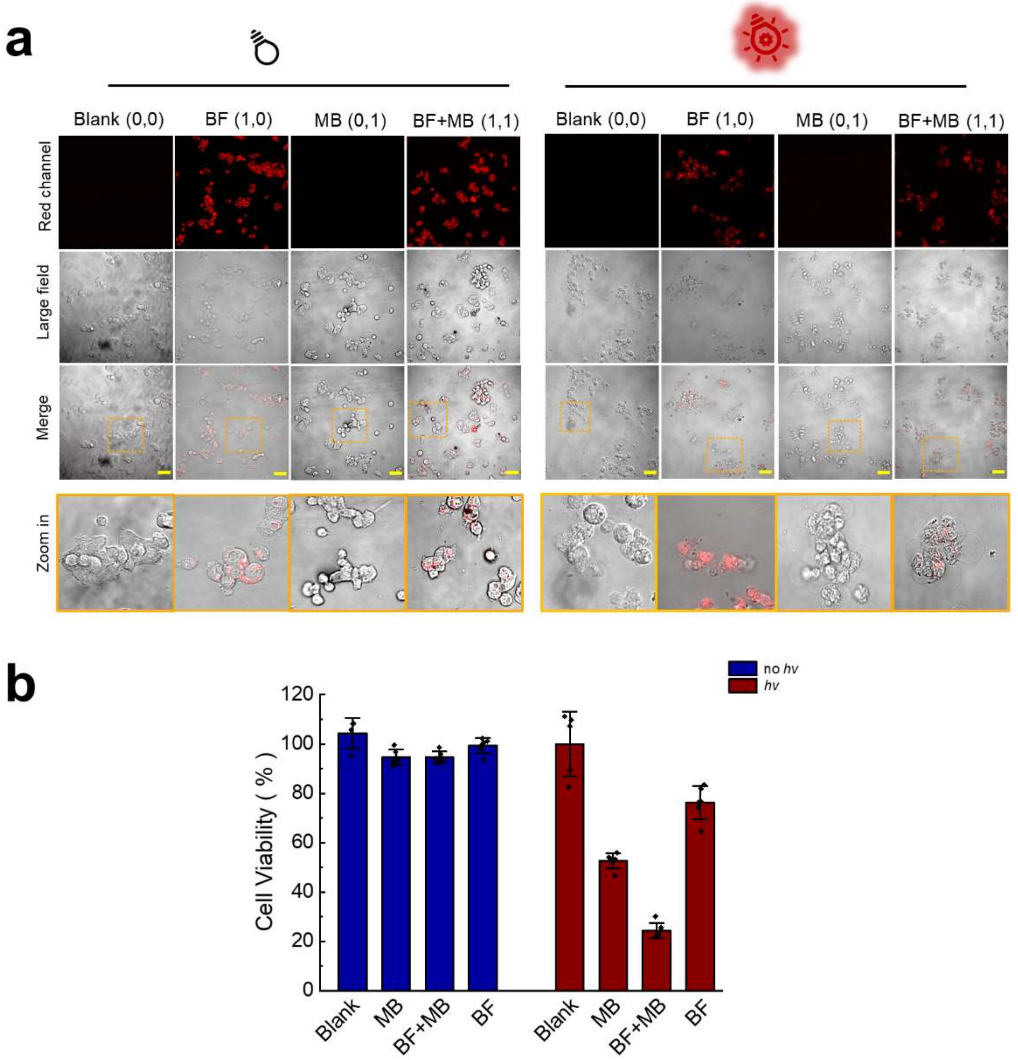

Figure S5. AND gated killing of HepG2 cell. **a**) Representative confocal microscopic images; scale bars: 50 μm. **b**) Statistical analysis of CCK-8 results.

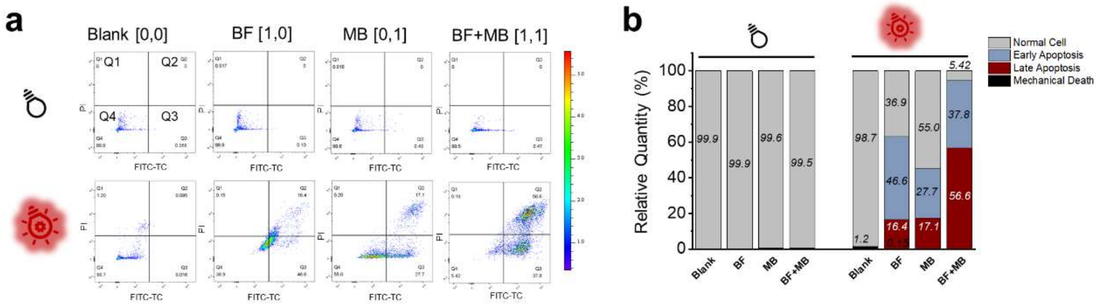

Figure S6. Mechanistic study shows that tumor cells undergo apoptosis. **a**) Flow cytometry analysis of fluorescein isothiocyanate (FITC) and propidium iodide (PI) co-labelled HeLa cells with or without illuminated with deep-red light (650 nm) operated by different logic gate inputs reveals that only in the presence of both drugs under red light illumination will late-stage apoptotic cells generate (Q2 quadrant, upright). **b**) Statistical quantification of the relative percentage of normal, early apoptotic live cell, late apoptotic died cell, and mechanical died cells is shown.

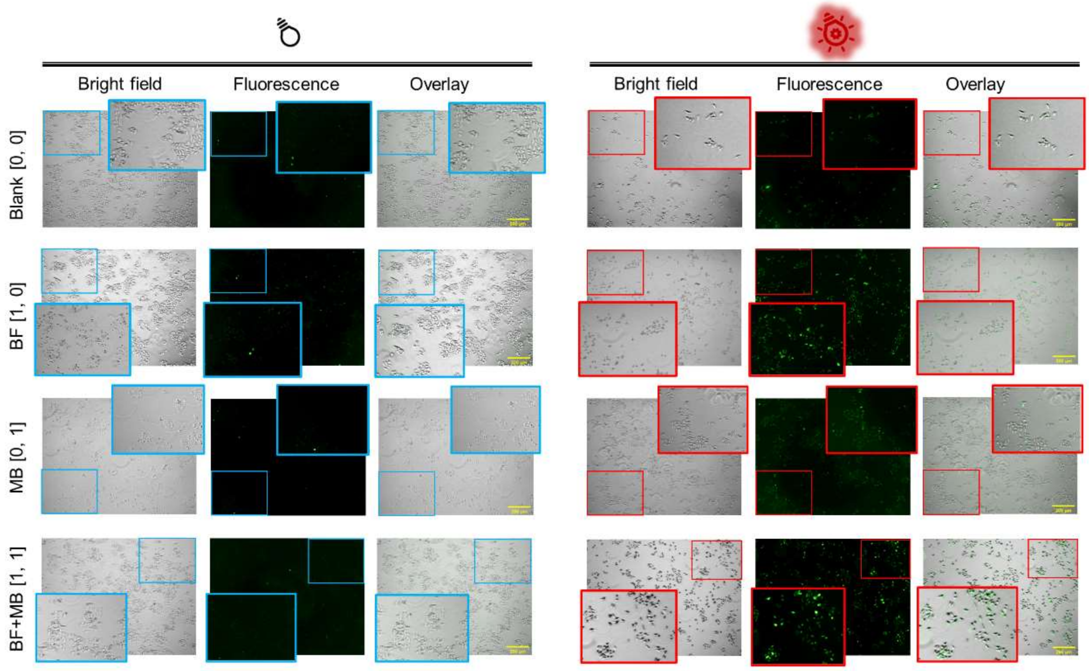

Figure S7. ROS was generated upon red light illumination in the presence of BF, MB, or BF+MB group. Confocal microscopic detection of the generation of ROS using DCFH-DA sensor reveals that BF [1, 0] at 2 μM, MB [0, 1] at 2 μM, or BF+MB [1,1] but not blank control [0,0] input cells show strong formation of ROS under 650 nm light illumination (right); a 50 W 650 nm red light lamp was used to illuminate cells on top of them (15 cm) for 30 min, and afterwards, the cell samples were subjected to fluorescence microscopic imaging. On the other hand, without light illumination, no ROS could be detected in all groups (left).

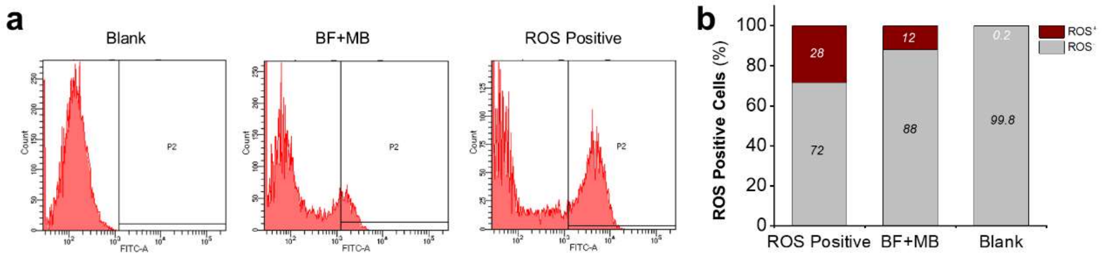

Figure S8. Further validation of ROS generation upon red light illumination in the BF+MB [1,1] group using flow cytometry analysis. **a**) Flow cytometry of HeLa cells treated with DCFH-DA sensor (λ_{ex} 488 nm/ λ_{em} 525 nm) reveals that BF+MB [1,1] input cells and ROS positive control cells generate ROS (P2 quadrant) but not the blank group cells. **b**) The ratio between normal cell numbers (P1) versus cells that generate ROS (P2) is statistically quantified.

Supplementary Methods

Ethical statement

Mice were maintained under specific pathogen-free (SPF) conditions and handled under the approval of the Institutional Animal Care and Use Committee (IACUC) of Harbin Institute of Technology with the permit number IACUC-2021052. Mice were housed under controlled light (12 h light / 12 h dark cycle), temperature (24 ± 2 °C), and humidity (50 ± 10%) conditions with free access to autoclaved chow and water. The maximal tumor size permitted by IACUC is 20 mm for the length or 10% of the body weight, and this limit was not exceeded in the study.

Generation of xenograft mouse model

The immunodeficient BALB/c nude female mice about 4-6 weeks were obtained from Beijing Vital River Laboratory Animal Technology Co., Ltd (Beijing, P.R. China). HepG2 cells were grown in a standard Φ ~ 85mm Petri dish to exponential phase before harvesting. Sex was not considered in the study design because hepatocarcinoma is not a gender-based disease. Then HepG2 cells were first washed by 10 ml PBS, added 1 ml of trypsin, digested for 5-10 min to allow full detachment of cells from the growing surface, and added 3 ml of PBS to suspend the detached cells. The cell suspension was subjected to centrifugation at 800× g for 8 min at 4 °C. The clear supernatant was discarded and the cell pellets were resuspended in a freshly prepared ice-cold 1:1 (v/v) mixture of PBS/Matrigel (Cat# M8370, Solarbio, Beijing, P.R. China) on ice. The final cell density is ~5×10^7 ml^{-1}. To make HepG2 xenograft mouse model, ~5×10^6 HepG2 cells in 0.1 ml of PBS/Matrigel solution were subcutaneously injected into the axillary region of the BALB/c nude mice. Tumor will typically appear in 1-2 weeks and will continue to grow steadily.

Mammalian cell culture

HeLa (Cat# CL-0101) and HepG2 (Cat# CL0103) cells were obtained from Procell Life Science & Technology Co., Ltd. (Wuhan, P.R. China), short tandem repeat (STR) identified and proven to be HIV-1, HBV, HCV, mycoplasma, and other microorganisms free before culturing. Other reagents such as full DMEM (Dulbecco's modified Eagle's medium) and PBS (phosphate buffered saline) were also confirmed to be mycoplasma free before usage. Unless otherwise specified, cell culture was maintained at 37°C under 5 % CO_2 in high glucose (4.5 $g\cdot L^{-1}$) DMEM (Cat# SH30243.01, HyClone, Logan, UT, USA) containing 4 mM L-glutamine and sodium pyruvate and supplemented with additional 10 % fetal bovine serum (FBS, Cat# SV30087.03, HyClone, Logan, UT, USA), 1 % non-essential amino acid (NEAA, 100×), and 1 % penicillin-streptomycin (100×). Trypsin-EDTA (Cat# SH30042.01, HyClone) and PBS (Cat# SH30256.01, HyClone, Logan, UT, USA) were used in subculturing. HeLa cells were subcultivated in a ratio of 1:5~10 while HepG2 cells were subcultivated in a ratio of 1:4~6.

Cell counting kit-8 (CCK-8) assay detects red light-induced phototoxicity.

For assessing red-light induced phototoxicity, HeLa or HepG2 cells were seeded in a 96-well UV-transparent plate with clear flat bottom (5×10^3 cells/well) and incubated at 37°C under 5% CO_2 overnight. 100 μl of drug solution at specified concentrations in full DMEM containing extra 15 mM 4-(2-hydroxyethyl)-1-piperazineethanesulfonic acid (HEPES) was added into each well for 1h in dark to allow drug uptake. The cells were exposed to a single-wavelength 650 nm deep red-light lamp at a distance of 15 cm on top for another

45 min. The cells were further incubated at 37 °C under 5% CO_2 in the dark for 2 h before subjected to CCK-8 cytotoxicity assay. For this, cells were washed three times by PBS, and then PBS was removed. A solution of 10 µl CCK-8 reagent (Cat# C0038, Beyotime Biotechnology, Shanghai, P.R. China) diluted in 90 µl full DMEM was added into each well and the cell culture plate was maintained in dark at 37°C under 5% CO_2 for 2 h before subjected to CCK-8 assay. For assessing dark cytotoxicity without red light illumination, drug solutions at specified concentrations in full DMEM containing extra 15 mM HEPES were added into each well of another 96-well plate seeded with the same number cells and incubated in dark for 3 h 45 min without any red-light illumination. All other parameters are the same as mentioned above. In CCK-8 assay, variable cells catalyze the conversion of the generally colorless WST-8 tetrazole to a yellow color WST-8 formazan that absorbs strongly at 450 nm. As a result, the relative absorptivity at 450 nm can indicate relative cell variability. Hence, the absorbance at 450 nm was measured using a multifunctional microplate reader (Tecan Infinite M200 pro) for calculation of cell viability.

$$\text{Cell Viability} = [(A_x - A_0)/(A_1 - A_0)] \times 100\%$$

where:

A_x is the absorbance of cells incubated with different drugs containing CCK-8 solutions;
A_0 is the absorbance of culture medium containing CCK-8;
A_1 is the absorbance of cells incubated with culture medium and CCK-8.

High performance liquid chromatography (HPLC) detects FTY720 release

dmBODIPY-FTY720 was first dissolved in DMSO (Cat# D103281, Aladdin, Shanghai, P.R. China) to form stock solution at 50mM concentration. Then, the stock solution was diluted in HPLC grade methanol (Cat# 67-56-1, Aladdin, Shanghai, P.R. China) to 100 µM in a total volume of 4 ml in a transparent glass bottle. dmBODIPY-FTY720 solution was subsequently subjected to illumination using a single-wavelength 650 nm deep red-light 50 W lamp source at a distance of 15 cm from side. At different time points (0 min, 10 min, 20 min, 40 min, 80 min, 120 min, 180 min, 360 min, 420 min), 200 µl of samples were taken and filtered through a 0.22 µm polytetrafluoroethylene (PTFE) membrane filter to remove any particles in order to avoid clogging of the liquid chromatography column. The filtrate was then placed into a micro-liquid sample bottle and each time 20 µl of sample was analyzed using Thermo Scientific™ Vanquish™ Core HPLC machine (Cat# VQF01-20001).

HPLC separation was performed through a Thermo Scientific™ Hypersil GOLD™ C18 column (column dimensions: 150 mm × 2.1 mm; Cat# 25005-152130). The mobile phase uses a combination of solvent A (0.05% TFA / H_2O) (Cat# C12077666, Macklin, Shanghai, P.R. China) and solvent B (0.05% TFA / ACN). Gradient elution at 1 $ml \cdot min^{-1}$ flow rate was employed in which the eluent composition increases linearly from 25 % B to 100 % B over 15 min and then continues for additional 3 min. Then, 100 % solvent B decreased linearly from 100% to 5% within 2 min. The peaks were detected using UV-Vis absorption at wavelengths of both 265 nm and 640 nm. Chameleon software program was used for peak analysis in the chromatograph, and the waterfall chromatograph was generated using Origin 2018 software (Origin Lab, Northampton, MA, USA).

Confocal microscopy

Live cells were imaged in phenol red free DMEM (Cat# 21063-29, Life Technologies, Carlsbad, CA, USA) supplemented with additional 10% FBS, 1% sodium pyruvate, 1%

NEAA, 1% penicillin-streptomycin and 15 mM HEPES at 37 °C under 5% CO_2. Microscopy was performed using NIKON A1HD25 inverted confocal laser scanning microscope. CFI Plan Apochromat Lambda 60X /N.A. 0.95 oil objective was primarily used for imaging while CFI Plan Fluor 40X (N.A. 0.75) oil objective was used as an alternative, for example, in recording larger field of views. Confocal images were typically acquired in 12-bit depth at 512 × 512 or 1024 × 1024 resolution. 561 nm laser diode was used to excite dmBODIPY-FTY720 or methylene blue. In most cases, typical parameters set as follows: scan speed 0.5, number of averaging 4, one-direction scanning, and pinhole 79.2 µm.

UV-vis absorption and fluorescence spectroscopic analysis of dmBODIPY-FTY720 and dmBODIPY

Stock solutions of dmBODIPY-FTY720 and dmBODIPY at 50 mM in DMSO (Cat# D103281, Aladdin, Shanghai, P.R. China) were first prepared and then diluted to 10 µM in PBS (Cat# BL302A, Biosharp Life sciences, Hefei, P.R. China). 100 µl of these PBS solutions were added into each well of a 96-well UV-transparent plate with clear flat bottom for measuring UV-Vis absorption. The absorption spectra data were recorded from 500 nm to 800 nm using a multi-functional microplate reader (Tecan Infinite M200 pro). Measurements were taken every 1 nm in step and repeated 5 times in each well to obtain an average value. PBS containing 0.02 %v DMSO was used as the blank.

Fluorescence spectra of the 10 µM PBS solution containing 0.02% DMSO were recorded in a quartz cuvette (d =1 cm, v =4 ml) using a fluorescence spectrometer (HORIBA Jobin Yvon). The excitation wavelength is 640 nm for dmBODIPY-FTY720 and 632 nm for dmBODIPY, and fluorescence measurements was taken every 1 nm in step.

Measurement of reactive oxygen species (ROS) generated upon drug/light treatment.

For assessing ROS level that is induced by red light illumination, Hela cells were seeded at a density of 6×10^4 cells/well in 6-well plate and incubated at 37°C under 5% CO_2 overnight. All subsequent treatments were carried out using DMEM cell culture medium containing extra 15 mM HEPES. Cells were treated with 5 µM dmBODIPY-FTY720 plus 2 µM MB (experimental group), DMEM (blank group), or ROSUP reagent (positive control group) from the Reactive Oxygen Species Assay Kit (Cat# S0033S, Beyotime Biotechnology, Shanghai, P.R. China) for 1 h in the dark to drug uptake. Then, the plate was exposed to a single-wavelength 650 nm deep red light 50 W lamp at a distance of 15 cm from top for additional 30 min, followed by 2 h incubation at 37°C under 5% CO_2. Afterwards, cells were stained using the Reactive Oxygen Species Assay Kit following the manufacturer's protocol. Briefly, cells were washed once in PBS and treated with a solution of 1 µM ROS-indicator dye DCFH-DA (2',7'-dichlorodihydrofluorescein diacetate) in DMEM for 20 min at 37°C in dark under 5% CO_2. After 20 min, the medium containing DCFH-DA dye was removed, and the cells were washed three times with PBS. ROS level was observed via green fluorescence using inverted fluorescence microscope (Olympus IX71) and analyzed using flow cytometry.

Flow cytometry analysis of drugs mechanism.

Analysis of apoptotic cells

After prior treatment as described in the study, cells were stained using the Annexin V-

FITC Apoptosis Detection Kit (Cat# C1062S, Beyotime Biotechnology, Shanghai, P.R. China) following the manufacturer's protocol. Briefly, cells were washed three times with PBS and then treated with 0.25 % trypsin-EDTA solution to detach the cells. Then the cell suspension was subjected to centrifugation at 3000 rpm for 5 min to obtain a cell pellet. The supernatant was discarded, and the cell pellet was gently washed once with cold PBS (pH 7.4) and then centrifuged again at 3000 rpm for 5 min. After discarding the PBS wash solution, the cell pellet was resuspended in 195 μl of 1× Annexin V binding buffer and thoroughly mixed. 5 μl fluorescein isothiocyanate (FITC) and 10 μl (propidium iodide (PI) solutions were added to each 195 μl of cell suspension. The cells were incubated in dark at room temperature for 15-20 min to finish staining, followed by the addition of 195 μl of binding buffer to each tube. The cell suspension was filtered through a 70 μm PTFE cell filter to prevent clogging of the flow cytometer. Flow cytometry was performed to analyze the stained cells using an excitation wavelength at 488 nm. The green emission of Alexa Fluor® 488 channel was recorded in the FL-1 channel, while the red emission of PI was recorded in the FL-3 channel. FlowJo software was used for data analysis (BD Bioscience, Ashland, OR, USA).

Analysis of ROS generation

After prior treatment as described in the study, HeLa cells were treated with fresh DMEM containing 1 μM DCFH-DA at 37 °C under 5% CO_2 for 20 min. Afterwards, the staining medium containing DCFH-DA dye was removed, and the cells were washed three times with PBS and then treated with 0.25% trypsin-EDTA solution to detach the cells. 0.5 ml of PBS was added and the cell suspension was filtered through a 70 μm PTFE cell filter to prevent clogging of the flow cytometer. The cell suspension was immediately subjected to flow cytometry analysis. The green color emission of the dye was detected using an excitation wavelength at 488 nm in the FL-1 channel.

Red light AND gated suppression of tumor growth *in vivo*

Drug solutions in DMSO were intratumorally injected into a tumor from different directions so that drugs could be distributed as evenly as possible inside a tumor. Unless otherwise specified, ~20 μl of dmBODIPY-FTY720 in DMSO (10.8 mg·ml^{-1}, final ~12 mg·kg^{-1}), ~20 μl of MB in DMSO (1.8 mg·ml^{-1}, final ~2 mg·kg^{-1} body weight), ~20 μl of dmBODIPY-FTY720 (10.8 mg·ml^{-1}) plus MB (1.8 mg·ml^{-1}) in DMSO ([1,1] AND input), or 20 μl of DMSO (blank, or [0, 0] input) was intratumorally given each time. Then, the tumor region was illuminated by 650 nm red light lamp for 60 min, and another 60 min of red-light illumination was applied the next day. This injection-illumination-illumination procedure was repetitively applied every two days. Tumor size data were recorded daily using a caliber [$\Phi = (\Phi_L + \Phi_S)/2$], and tumor volumes were calculated using the following equation: $V = 1/6(\pi\Phi^3)$.

Imaging of dmBODIPY-FTY720 activation *in vivo*.

For imaging of fluorescence in vivo, a mouse was first injected with ~20 μl of dmBODIPY-FTY720 (10.8 mg·ml^{-1} in DMSO, final ~12 mg·kg^{-1} of body weight), and then gas-anesthetized using isoflurane (Cat# R510-22-10, RWD Life science, Shenzhen, P.R. China) while oxygen was used as carrier gas at a flow rate of 1.5 lpm within 1 min until the mouse stopped to move. Then the mouse was illuminated by a single wavelength 650 nm lamp at

a distance of 15 cm on top for varying durations of time (0 min, 5 min, 10 min, 20 min, 35 min, and 60 min). After each illumination interval, the mouse was imagined using a living imaging system for small animals (PerkinElmer IVIS Lumina XRMS) excited at 620 nm while recorded at 670 nm channel.

Organic Synthesis

General

Unless otherwise specified, all chemicals were purchased from commercial vendors and were used without further purification. The ^{1}H- and ^{13}C-NMR spectra were measured on a 600 MHz Bruker BioSpin GmbH magnetic resonance spectrometer. Data for ^{1}H-NMR spectra are reported as follows: Chemical shifts are reported as δ in units of parts per million (ppm); multiplicities are reported as follows: s (singlet), d (doublet), t (triplet), q (quartet), dd (doublet of doublets), m (multiplet), or br (broadened); coupling constants are reported as J values in Hertz (Hz); the number of protons (n) for a given resonance is indicated as nH, and is based on the spectra integration values. High resolution mass spectra (HR-MS) measurement was performed via a customer service using electron spray ionization (ESI).

Overall synthetic scheme

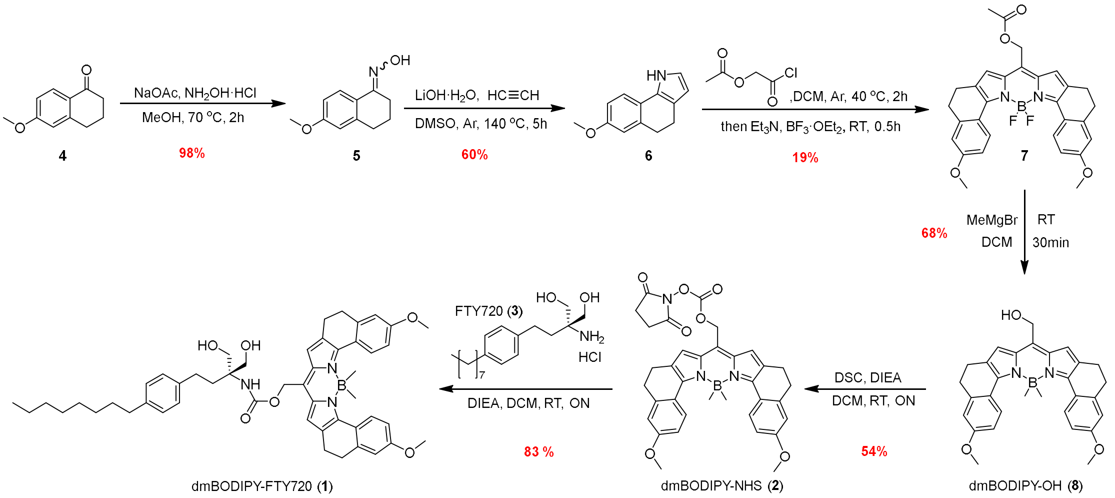

Scheme S1. Synthetic scheme for the preparation of dmBODIPY-FTY720 photocaged immunosuppressant. Abbreviations: DMSO, dimethyl sulfoxide; DCM, dichloromethane; Et_3N, triethylamine; DSC, *N,N'*-disuccinimidyl carbonate; DIEA, *N,N*-diisopropylethylamine; FTY720, 2-amino-2-[2-(4-octylphenyl)ethyl]propane-1,3-diol hydrochloride.

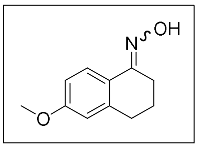

6-Methoxy-3,4-dihydronaphthalen-1(2*H*)-one oxime (5)

Into a thoroughly dried 250 ml two-necked round bottom flask (RBF), 6-methoxy tetralone (10.0 g, 56.8 mmol), sodium acetate (5.6 g, 68.2 mmol), hydroxylamine hydrochloride (4.7 g, 68.2 mmol), and anhydrous methanol (40 ml) were added. The mixture was then stirred and heated to reflux at 70°C for 1.5 h. Afterwards, the reaction solution was allowed to cool down to room temperature, concentrated, and treated with 70 ml of H_2O. The resulting suspension was filtered via suction filtration and the resulting cake washed with 70 ml of deionized water and then 40 ml of cyclohexane to give the product without further purifications. The solid was vacuum dried to yield 10.1 g light orange granular solid product in a yield of 93%. **^{1}H-NMR** (600 MHz, $CDCl_3$) δ 8.82 (s, 1H), 7.82 (d, J = 8.8 Hz, 1H), 6.78 (d, J = 2.7 Hz, 1H), 6.66 (s, 1H), 3.82 (s, 3H), 2.81 (t, J = 7.0 Hz, 2H), 2.74 (t, J = 6.1 Hz, 2H), 1.88 (p, J = 6.0 Hz, 2H). **^{13}C-NMR** (151 MHz, $CDCl_3$) δ 160.30, 155.17, 141.56, 125.68,

123.29, 113.02, 112.91, 55.26, 30.14, 24.22, 20.97. **HRMS**: $C_{11}H_{14}NO_2^+$ [M+H]$^+$ calcd.192.1019, found 192.1024.

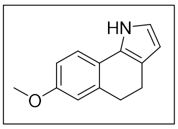

7-Methoxy-4,5-dihydro-1*H*-benzo[*g*]indole (6)

First, in order to produce acetylene, CaC_2 (22 g, 98 %) was placed into a dried 250 ml two-necked RBF cooled in ice water. Saturated saline (50 ml) was dropwise added onto CaC_2 solid to smoothly generate crude acetylene gas. The rate of addition was carefully controlled at around 20-30 seconds per drop to ensure the gentle and stable generation of acetylene gas. The resulting gas was further purified and dried by passing through 20% copper sulfate solution and a U-shaped tube filled with soda lime. This process produced acetylene gas with sufficient quality for subsequent cyclization reactions.

At the same time, the acetylene gas was bubbled into a suspension of lithium hydroxide monohydrate (1.0 g, 23.8 mmol), and above prepared oxime intermediate **5** (1.0 g, 5.2 mmol) in 25 ml DMSO at 140 °C in a thoroughly dried 250 ml two-necked RBF. The reaction was allowed to proceed at 140 °C for 5 h. Since the reactant can be rapidly oxidized in air with the formation of blue color species, thin layer chromatography (TLC) can be used to easily monitor the proceeding of the reaction by visualization of the blue spot on the TLC plate. 5h later, the reaction solution was cooled down and water (250 ml) was slowly added to quench the reaction mixture. The aqueous phase was separated and extracted with ethyl acetate (3×100 ml). The combined organic fractions were washed with brine (100 ml), dried over anhydrous Na_2SO_4, filtered, concentrated, and purified via silica gel flash chromatography (cyclohexane/DCM 5:1→1:1) to give 561 mg white solid as the pyrrole intermediate in a yield of 54%. **^{1}H-NMR** (600 MHz, $CDCl_3$) δ 8.25 (s, 1H), 7.08 (d, J = 8.3 Hz, 1H), 6.72 (d, J = 11.2 Hz, 2H), 6.11 (s, 1H), 3.81 (s, 3H), 2.92 (t, J = 7.8 Hz, 2H), 2.74 (t, J = 7.9 Hz, 2H).**^{13}C-NMR** (151MHz, $CDCl_3$,): δ 157.47, 136.95, 127.85, 123.27, 119.33, 118.48, 117.37, 114.80, 111.35, 108.07, 55.44, 30.67, 21.94; **HRMS**: $C_{13}H_{14}NO^+$ [M+H]$^+$ calcd. 200.1070, found 200.1073.

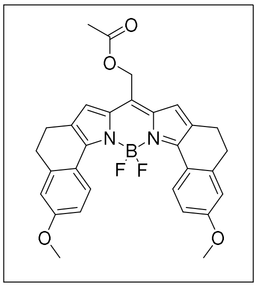

***B*, *B*-Difluoro-BODIPY-methyl acetate (dfBODIPY-OAc, 7).**

Into a 10 ml completely dried two-necked RBF, acetoxy acetyl chloride (0.14 ml, 1.3 mmol) was added to a solution of pyrrole intermediate **6** (0.4 g, 2.0 mmol) in anhydrous DCM (1.6 ml, 0.25 M). The mixture was stirred for 2 h under a nitrogen atmosphere at 40 °C. After the reaction was cooled to room temperature, triethylamine (0.73 ml, 4.2 mmol) was added, followed by the dropwise addition of $BF_3{\cdot}OEt_2$ (0.53 ml, 4.2 mmol). After being stirred for a further 0.5 h, the solvent was removed under reduced pressure, and the crude product was purified by flash chromatography through a silica gel column (cyclohexane /DCM=1:1). The product was further purified via recrystallization using DCM/ cyclohexane to obtain 201 mg

BODIPY intermediate **7** as a dark green metal glossy solid in a yield of 19%.**^{1}H-NMR** (600 MHz, $CDCl_3$) *δ* 8.72 (d, *J* = 8.9 Hz, 2H), 7.00 – 6.95 (m, 4H), 6.81 (d, *J* = 2.7 Hz, 2H), 5.25 (s, 2H), 3.87 (s, 6H), 2.90 (t, *J* = 7.0 Hz, 4H), 2.74 (t, *J* = 7.1 Hz, 4H), 2.11 (s, 6H). **^{13}C-NMR** (151 MHz, $CDCl_3$) *δ* 169.54, 159.81, 151.49, 142.03, 135.44, 131.43, 129.23, 120.96, 120.31, 113.30, 111.05, 58.72, 53.59, 29.85, 28.67, 21.40, 19.54. **HRMS**: $C_{30}H_{27}BF_2N_2NaO_4^+$ [M+Na]$^+$ calcd. 551.1924, found 551.1937

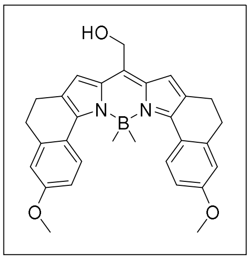

***B*, *B*-Dimethyl BODIPY-methyl alcohol (dmBODIPY, 8).**

A solution of dfBODIPY intermediate **7** (40 mg, 0.076 mmol) in 2.5 ml of anhydrous DCM was prepared in a completely dried 10 ml two-necked RBF, followed by the slow dropwise addition of 375 µl of 3 M magnesium bromide methylate (4.5 mmol) to the reaction mixture. The reaction was stirred at room temperature under nitrogen and monitored on the TLC. After completion of the reaction, the mixture was diluted in 5 times the volume of DCM (50 ml). To quench the reaction, 2.5 ml of saturated ammonium chloride was added, and the organic phase was collected. The combined organic fractions were washed with brine, dried (Na_2SO_4), filtered, concentrated, and purified via flash chromatography on a silica gel column (cyclohexane/ ethyl acetate 4:1) to obtain 21. 8 mg product **8** as a blue-green solid with a yield of 60%. **^{1}H-NMR** (600 MHz, $CDCl_3$) *δ* 8.26 (d, *J* = 8.8 Hz, 2H), 6.95 (s, 2H), 6.84 (dd, J^1 = 8.9, J^2=2.8 Hz, 2H), 6.74 (d, *J* = 2.8 Hz, 2H), 4.81 (s, 2H), 3.80 (s, 6H), 2.77 (t, *J* = 6.8 Hz, 4H), 2.67 (t, *J* = 7.0 Hz, 4H), 0.50 (s, 6H). **^{13}C-NMR** (151 MHz, $CDCl_3$) *δ* 159.14, 149.71, 142.75, 134.48, 133.61, 132.36, 131.15, 122.56, 118.67, 114.07, 110.67, 60.42, 55.29, 31.45, 29.71, 23.00, 21.08. **HRMS**: $C_{30}H_{32}BN_2O_3^+$ [M+H]$^+$ calcd. 479.2500, found 479.2491.

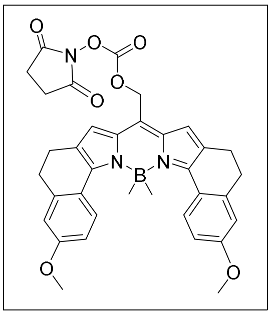

***B*, *B*-Dimethyl BODIPY- *N,N'*-disuccinimidyl carbonate (dmBODIPY-NHS, 2)**

In a completely dried 2 ml centrifucation tube, a solution of compound **8** (10 mg 0.021 mmol) and *N,N'*-disuccinimidyl carbonate (16.1 mg, 0.063 mmol) in 418 µl of anhydrous DCM was prepared, followed by the addition of *N, N*-divisopropylethylamine (17.24 µl, 0.10 mmol) to the reaction mixture. The reaction was stirred at room temperature for 30 min under nitrogen and monitored on the TLC. Once completed, the combined organic fractions were neutralized with saturant NaH_2PO_4, washed with brine, dried (Na_2SO_4), filtered, concentrated, and purified via silica gel chromatography (cyclohexane/ ethyl acetate 5:1→1:1) to obtain 7.0 mg compound **2** as an Indigo solid with a yield of 54%.**^{1}H-NMR** (600

MHz, $CDCl_3$) δ 8.26 (d, J = 8.9 Hz, 2H), 6.86 (s, 2H), 6.84 (dd, J^1= 8.9, J^2=2.8 Hz, 2H), 6.73 (s, 2H), 5.46 (s, 2H), 3.80 (s, 6H), 2.79 – 2.76 (m, 8H), 2.69 – 2.66 (m, 4H), 0.50 (s, 6H). **^{13}C-NMR** (151 MHz, $CDCl_3$) δ 168.46, 159.82, 143.08, 133.70, 132.99, 131.39, 124.71, 122.37, 118.90, 114.10, 111.95, 67.47, 53.89, 31.01, 26.93, 25.49, 22.98. **HRMS**: $[M+Na]^+$ $C_{35}H_{34}BN_3NaO_7^+$ calcd. 642.2382, found 642.2379.

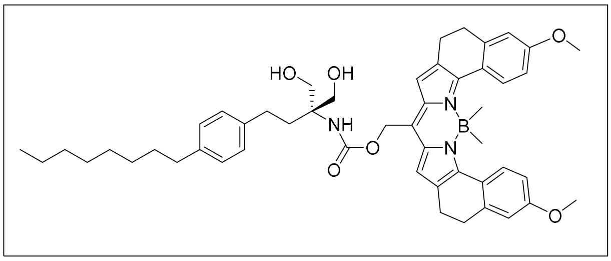

***B,B*-Dimethyl BODIPY-N-FTY720 (dmBODIPY-FTY720, 1)**

In a completely dried 2 ml EP tube, dmBODIPY-OH(4 mg, 0.006 mmol), fingolimod (2.06 mg, 0.006 mmol), and anhydrous DCM (120 µL, 0.05 M) were added. The reaction was then stirred under nitrogen for 3 h and monitored on the TLC. Once completed, the combined organic fractions were neutralized with saturant NaH_2PO_4, washed with brine, dried (Na_2SO_4), filtered, concentrated, and purified via flash chromatography on a silica column (cyclohexane/ ethyl acetate 5:1→1:1) to obtain 4.0 mg product **1** as an Indigo solid with a yield of 81%.**^{1}H NMR** (600 MHz, $CDCl_3$) δ 8.33 (d, J = 8.8 Hz, 2H), 7.10 – 7.06 (m, 4H), 7.00 (s, 2H), 6.91 (dd, J^1= 8.9, J^2=2.8 Hz, 2H), 6.80 (d, J = 2.8 Hz, 2H), 6.06 (s, 1H), 5.42 (s, 1H), 5.32 (s, 2H), 3.92 (d, J = 8.8 Hz, 2H), 3.87 (s, 6H), 3.69 (d, J = 7.1 Hz, 2H), 3.63 (d, J = 11.5 Hz, 1H), 3.53 (d, J = 11.5 Hz, 1H), 2.81 (t, J = 6.7 Hz, 4H), 2.74 – 2.69 (m, 4H), 2.62 (t, J = 8.4 Hz, 2H), 2.57 – 2.54 (m, 3H), 1.95 – 1.89 (m, 3H), 1.58 – 1.54 (m, 2H), 1.30 – 1.27 (m, 11H), 0.88 (s, 3H), 0.58 (s, 6H). **^{13}C NMR** (151 MHz, $CDCl_3$) δ 159.59, 149.38, 142.79, 140.94, 138.45, 137.56, 134.17, 132.51, 131.16, 128.58, 128.43, 128.02, 127.98, 122.38, 118.98, 113.99, 111.11, 70.65, 66.36, 66.22, 61.65, 61.32, 60.36, 59.64, 55.19, 37.03, 35.45, 31.82, 31.48, 31.32, 29.63, 29.40, 29.29, 29.20, 29.14, 28.98, 22.89, 22.20, 14.05. **HRMS**: $[M+Na]^+$ $C_{50}H_{62}BN_3NaO_6^+$ calcd. 834.4624, found 834.4620.

NMR Spectra

^{1}H NMR (600MHz, $CDCl_3$): 6-Methoxy-3,4-dihydronaphthalen-1(2*H*)-one oxime

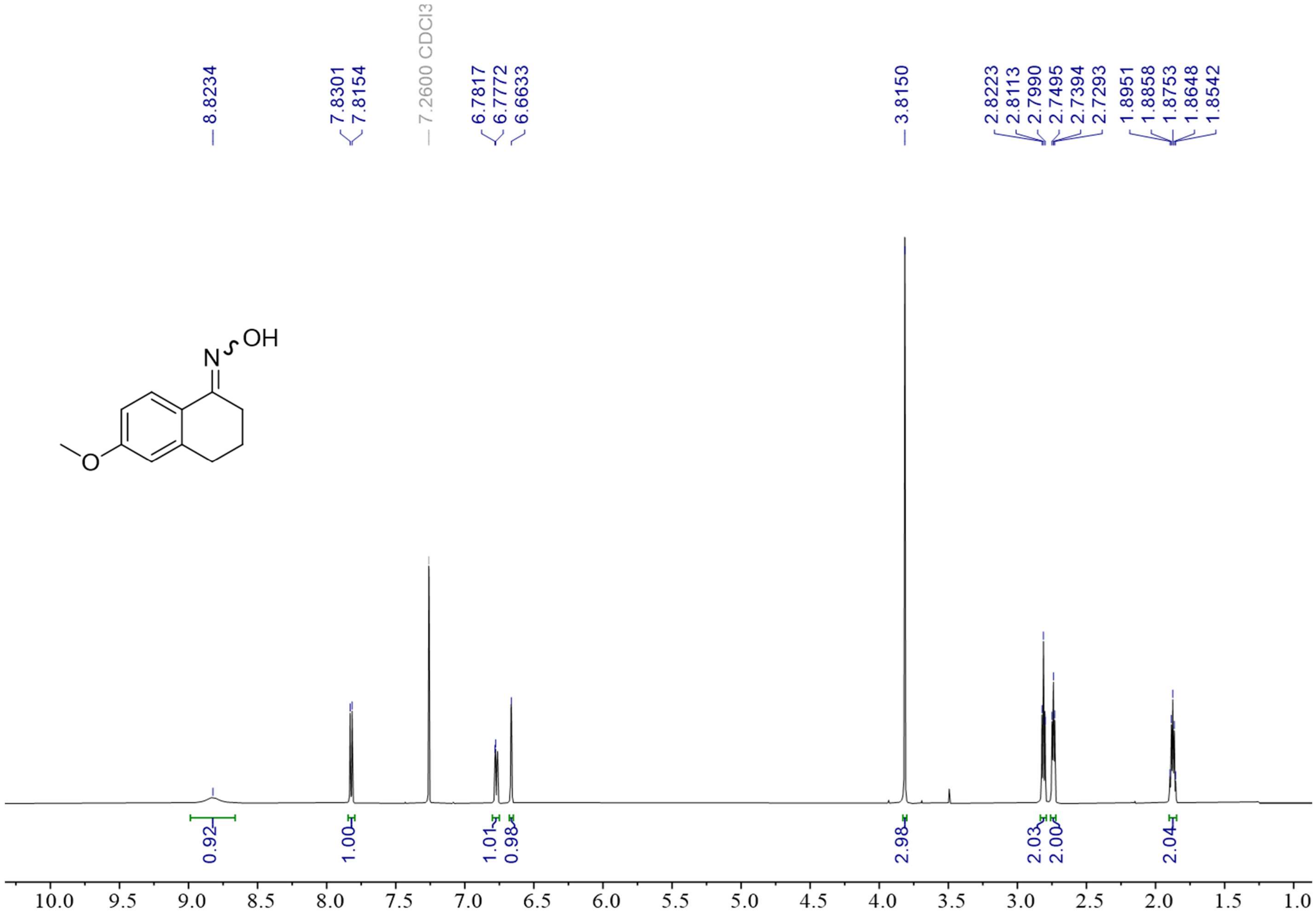

^{13}C NMR (151MHz, $CDCl_3$): 6-Methoxy-3,4-dihydronaphthalen-1(2*H*)-one oxime

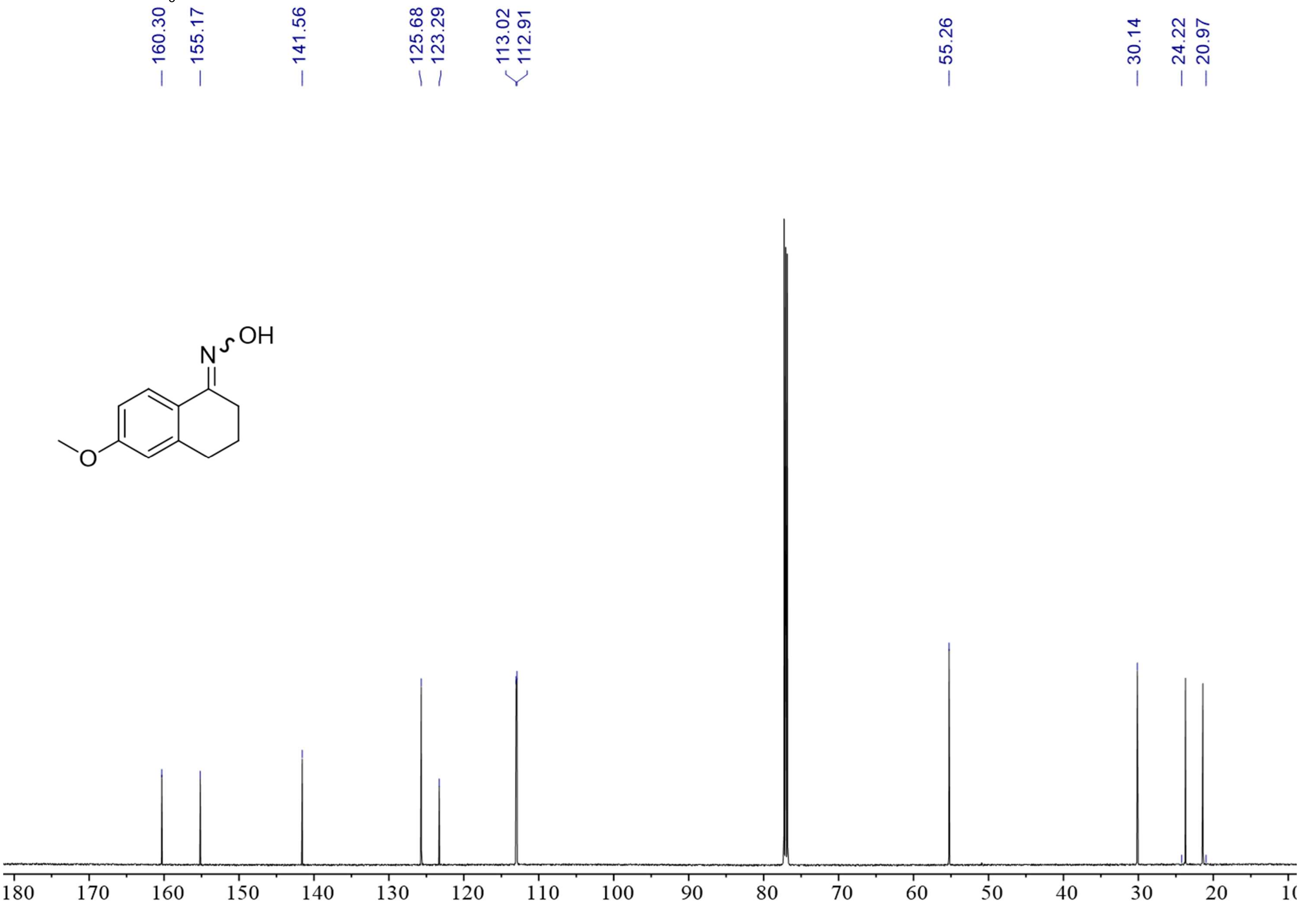

^{1}H NMR (600MHz, $CDCl_3$): 7-Methoxy-4,5-dihydro-1H-benzo[g]indole

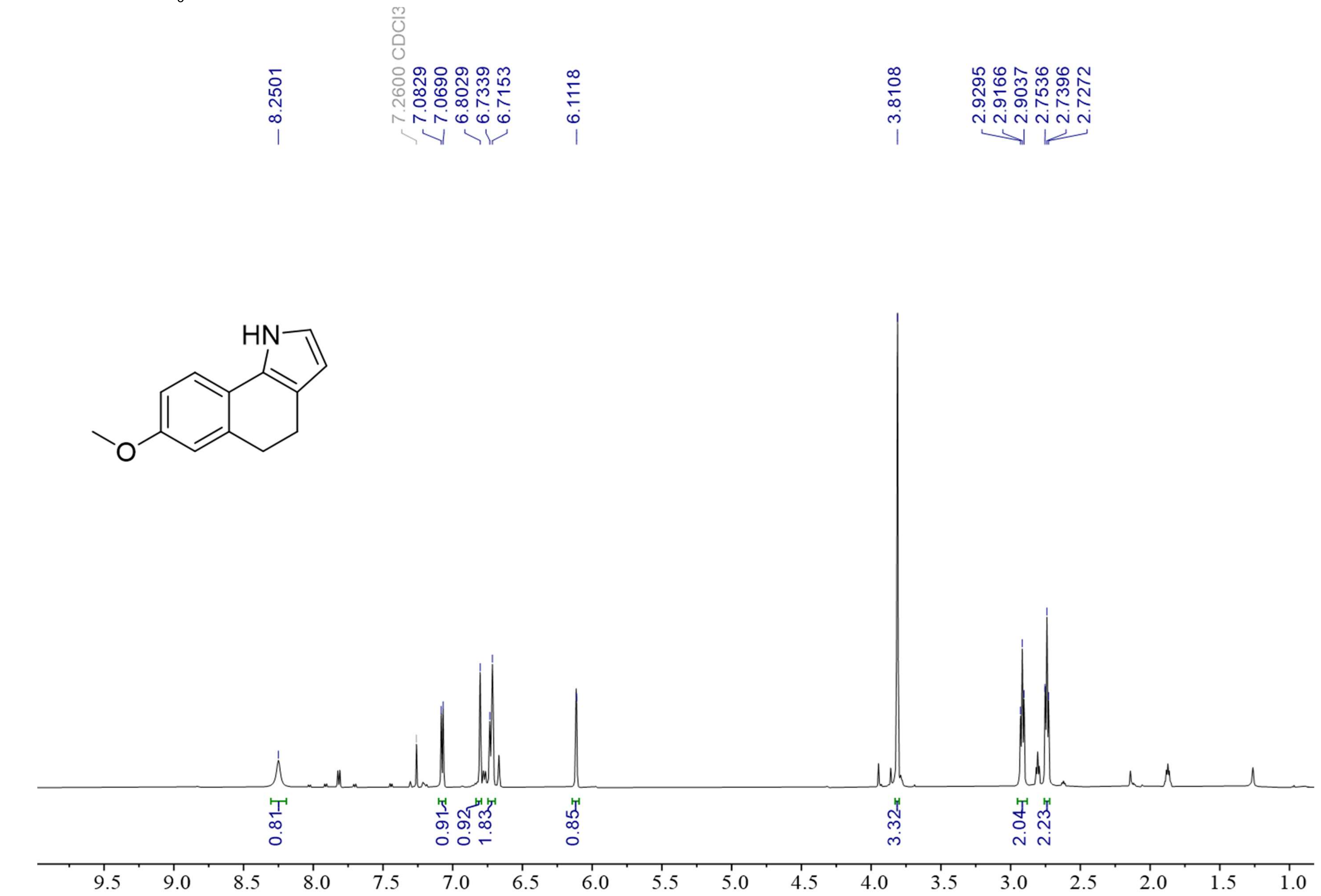

^{13}C NMR (151MHz, $CDCl_3$): 7-Methoxy-4,5-dihydro-1H-benzo[g]indole

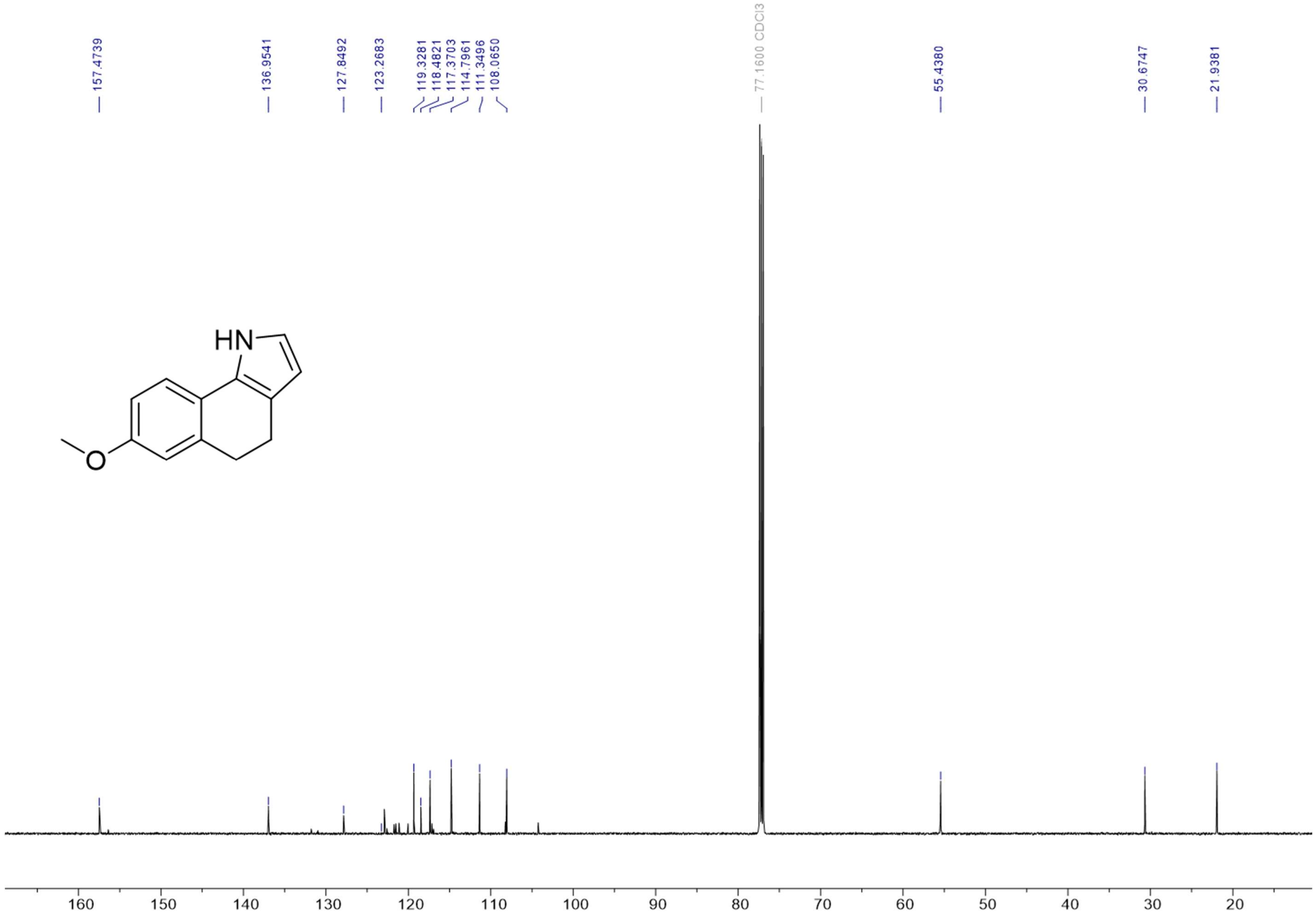

¹H NMR (600MHz, $CDCl_3$): *B*, *B*-difluoro-BODIPY-methyl acetate

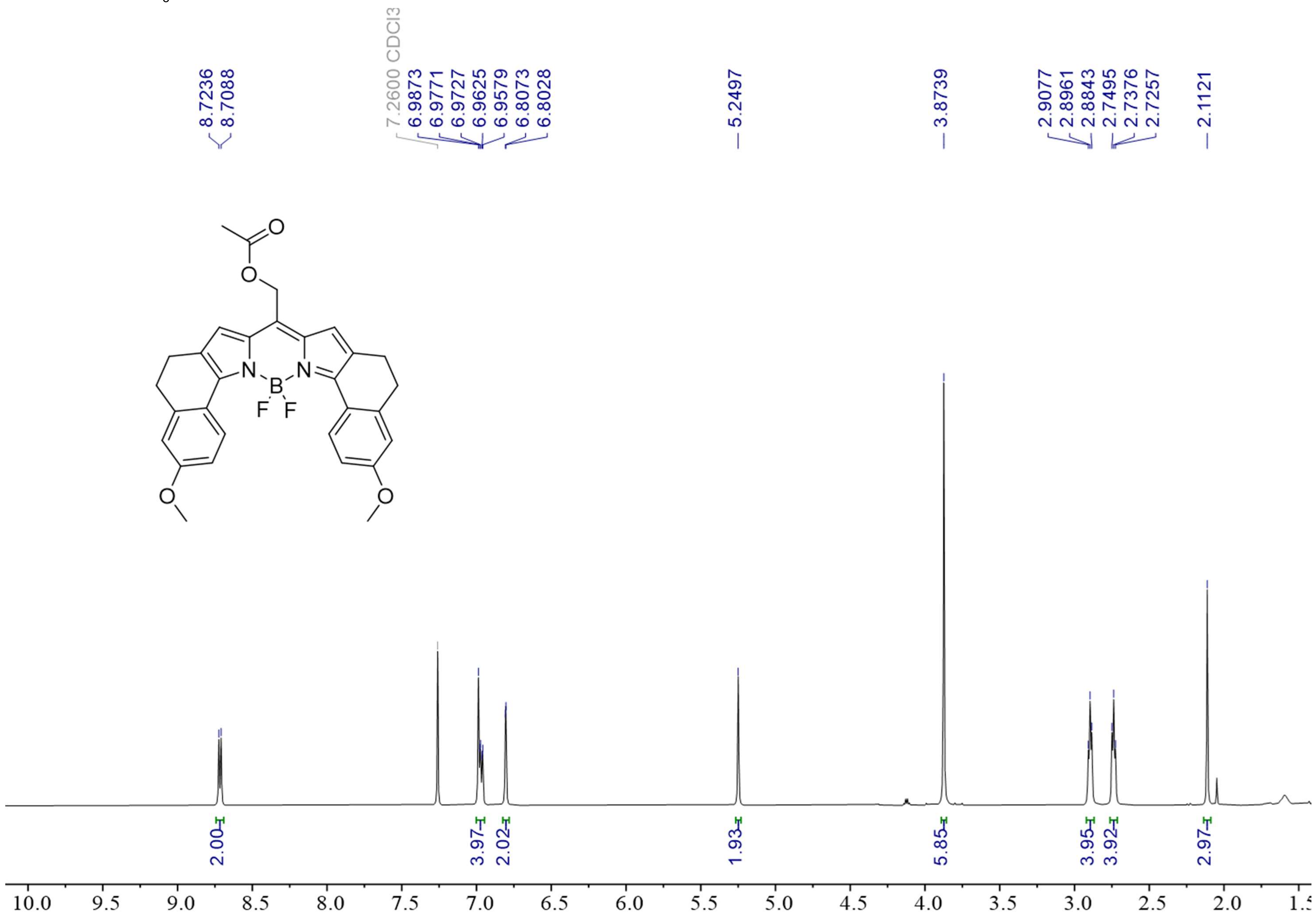

^{13}C NMR (151MHz, $CDCl_3$): *B*, *B*-difluoro-BODIPY-methyl acetate

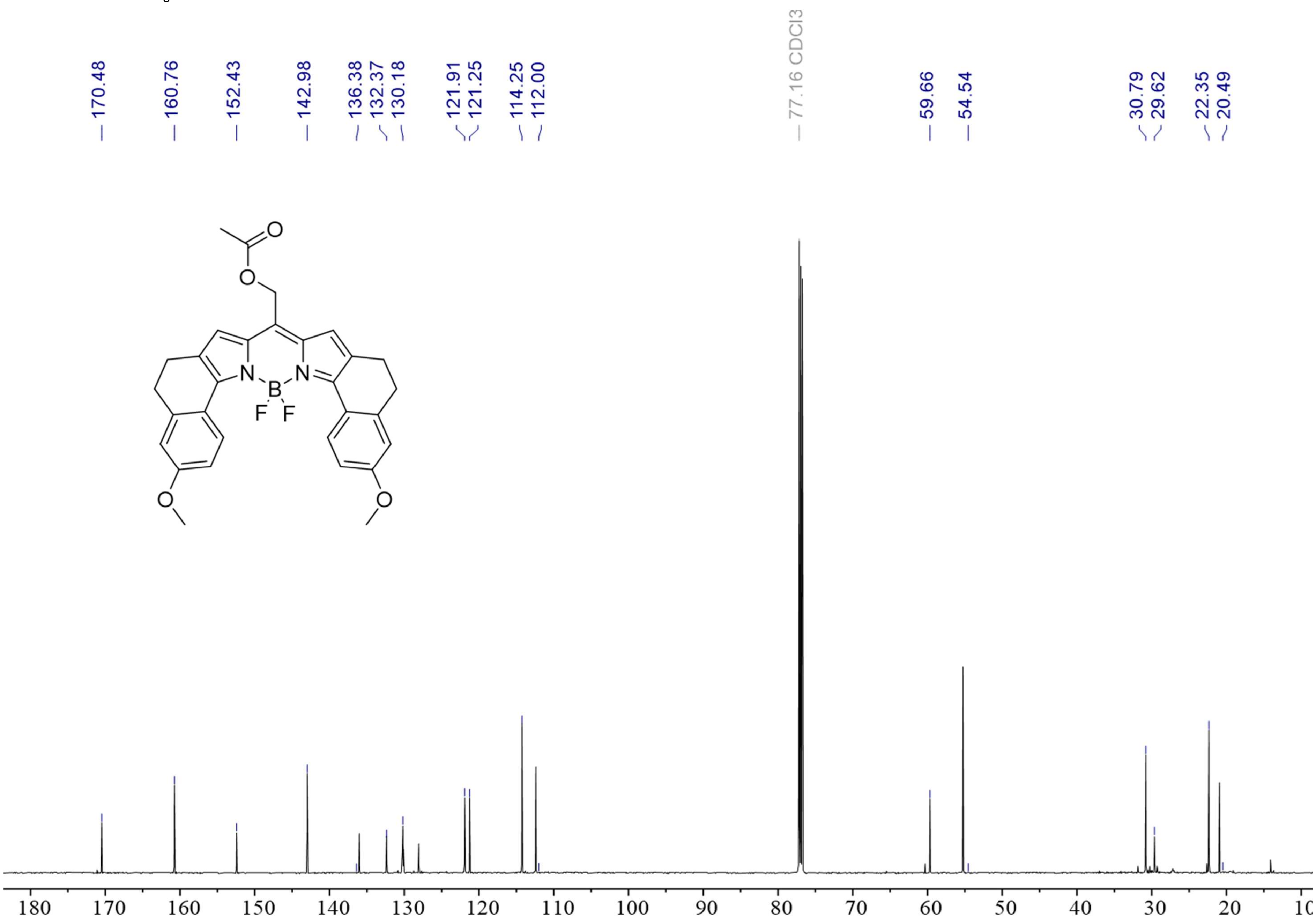

^{1}H NMR (600MHz, $CDCl_3$): *B, B*-dimethyl BODIPY-methyl alcohol

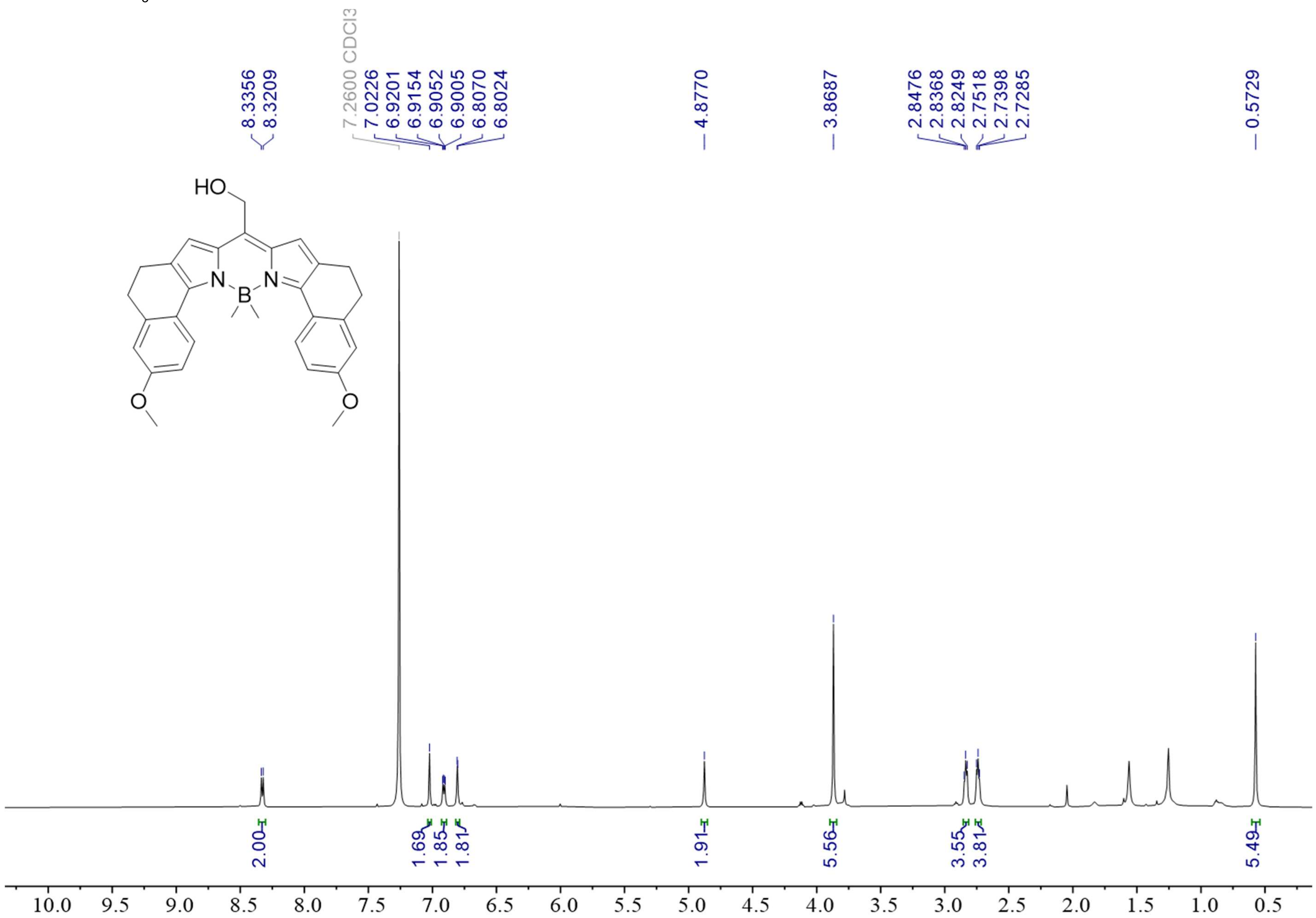

^{13}C NMR (151MHz, $CDCl_3$): *B, B*-dimethyl BODIPY-methyl alcohol

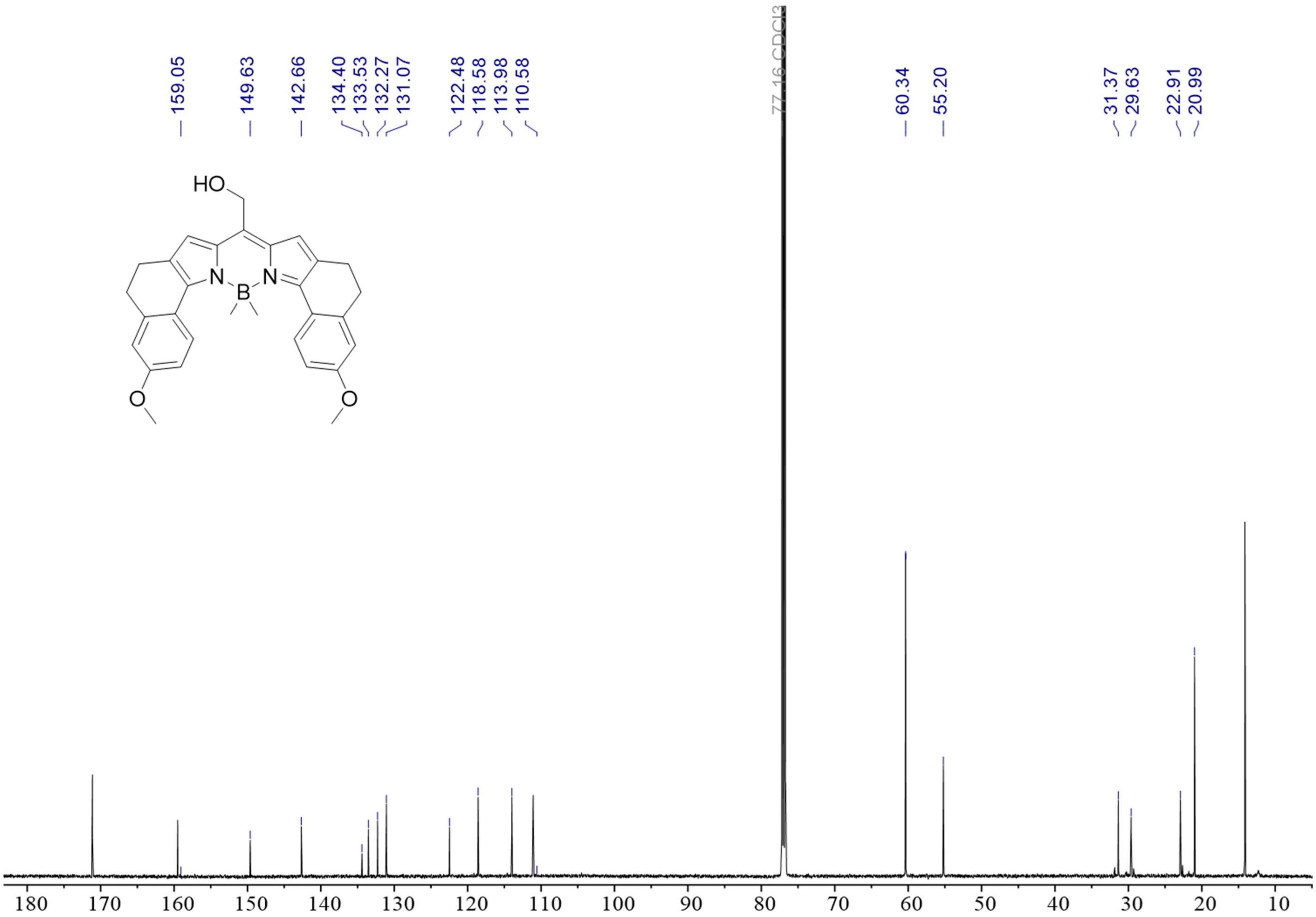

¹H NMR (600MHz, $CDCl_3$): *B, B*-Dimethyl BODIPY-N-succinimidyl carbonate

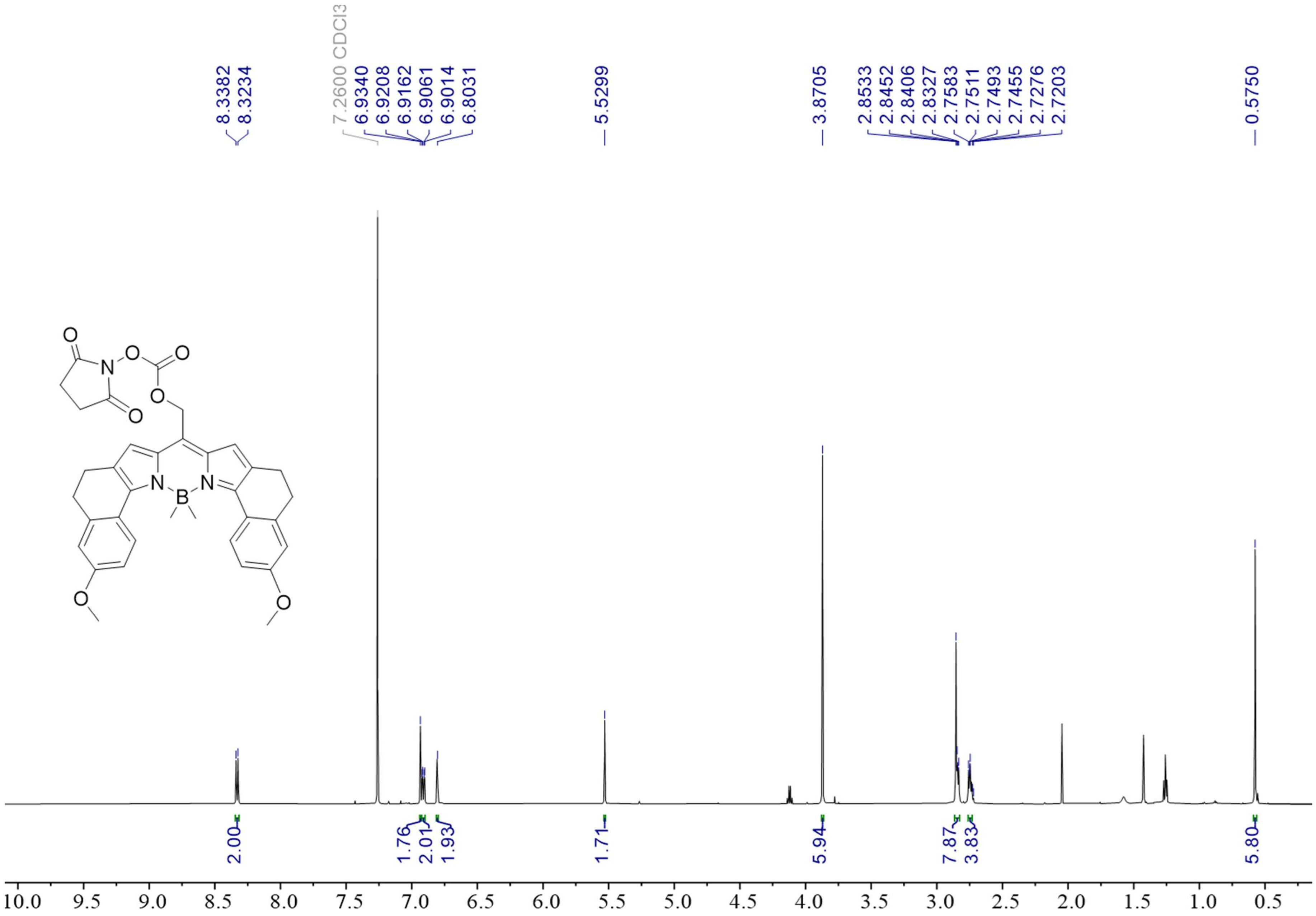

^{13}C NMR (151MHz, $CDCl_3$): *B, B*-Dimethyl BODIPY-N-succinimidyl carbonate

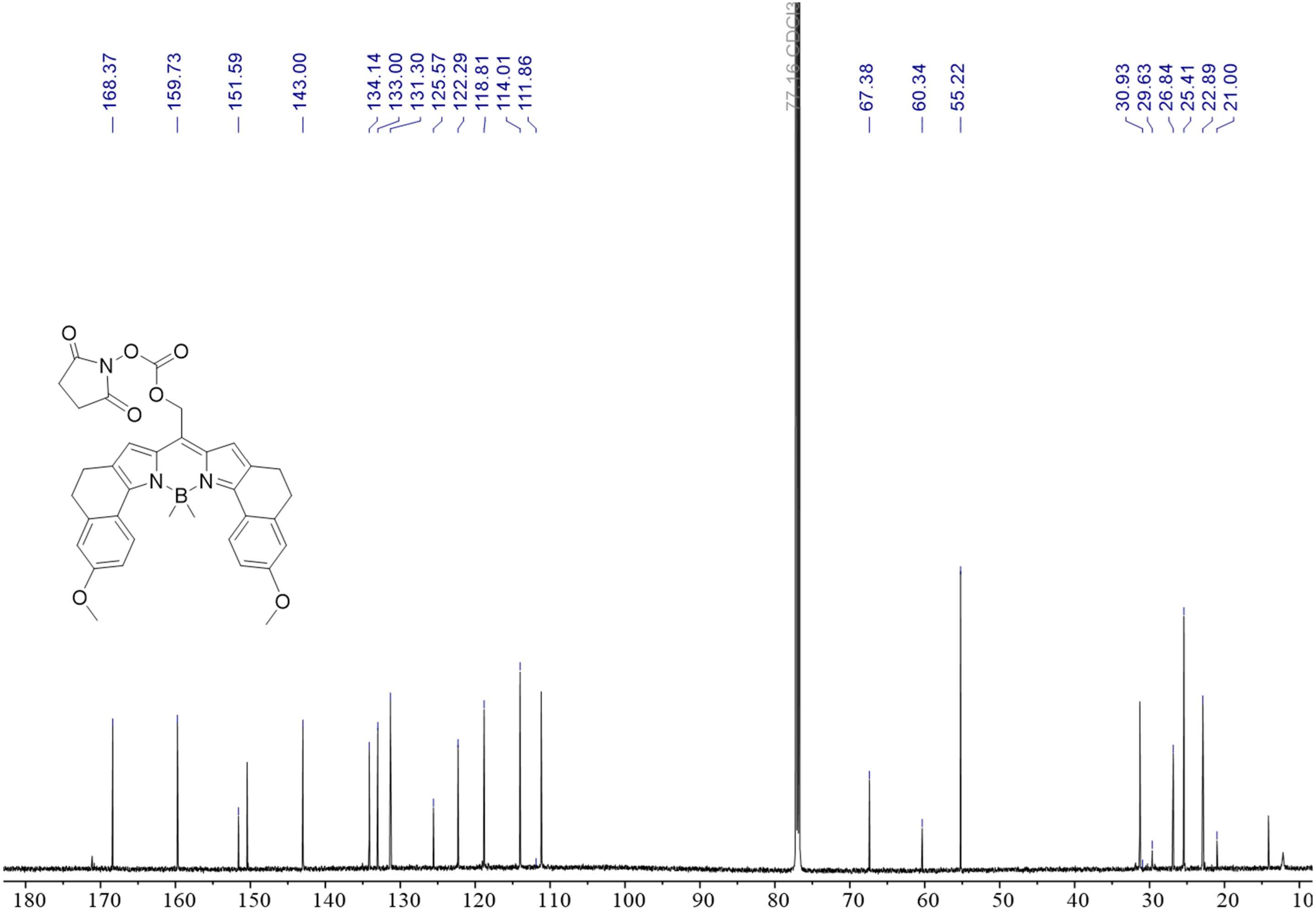

¹H NMR (600MHz, CD_3OD): *B,B*-Dimethyl BODIPY-N-FTY720

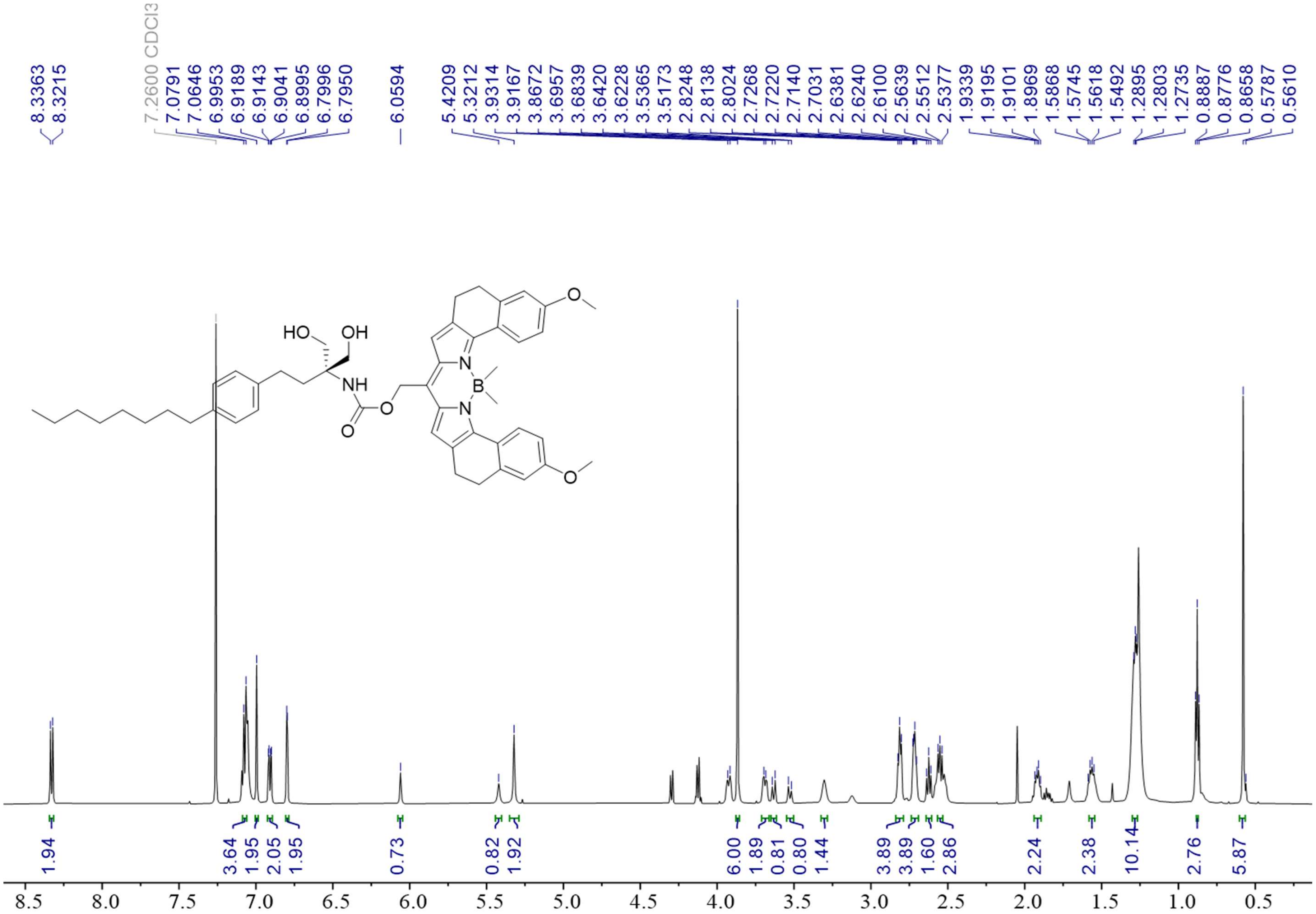

^{13}C NMR (151MHz, $CDCl_3$): *B,B*-Dimethyl BODIPY-N-FTY720

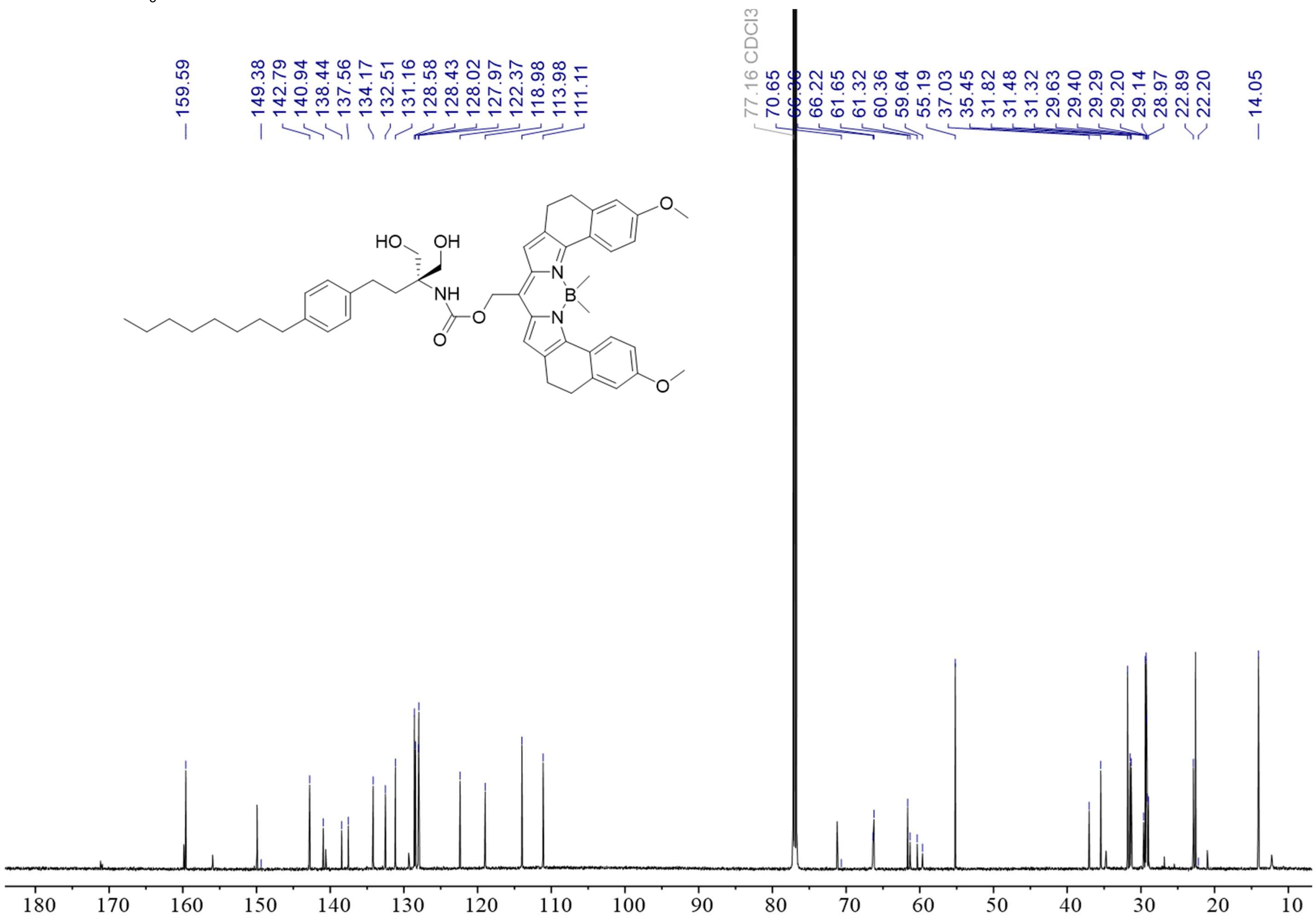

Supplement: Supplementary file 1 [file cells-12-02351-s001.zip › cells-2528607-supplementary.pdf]
